# Functional Peroxisomes Are Essential for Efficient Cholesterol Sensing and Synthesis

**DOI:** 10.3389/fcell.2020.560266

**Published:** 2020-11-06

**Authors:** Khanichi N. Charles, Janis E. Shackelford, Phyllis L. Faust, Steven J. Fliesler, Herbert Stangl, Werner J. Kovacs

**Affiliations:** ^1^Department of Biology, San Diego State University, San Diego, CA, United States; ^2^Department of Pathology and Cell Biology, Vagelos College of Physicians and Surgeons, Columbia University, New York, NY, United States; ^3^Departments of Ophthalmology and Biochemistry and Gradate Program in Neuroscience, University at Buffalo-The State University of New York (SUNY), Buffalo, NY, United States; ^4^Research Service, Veterans Administration Western New York Healthcare System, Buffalo, NY, United States; ^5^Department of Medical Chemistry, Center for Pathobiochemistry and Genetics, Medical University of Vienna, Vienna, Austria; ^6^Institute of Molecular Health Sciences, ETH Zurich, Zurich, Switzerland

**Keywords:** cholesterol synthesis, CHO cells, ER-to-Golgi transport, peroxisomes, PEX2, SCAP, SREBP-2, Zellweger syndrome

## Abstract

Cholesterol biosynthesis is a multi-step process involving several subcellular compartments, including peroxisomes. Cells adjust their sterol content by both transcriptional and post-transcriptional feedback regulation, for which sterol regulatory element-binding proteins (SREBPs) are essential; such homeostasis is dysregulated in peroxisome-deficient *Pex2* knockout mice. Here, we compared the regulation of cholesterol biosynthesis in Chinese hamster ovary (CHO-K1) cells and in three isogenic peroxisome-deficient CHO cell lines harboring *Pex2* gene mutations. Peroxisome deficiency activated expression of cholesterogenic genes, however, cholesterol levels were unchanged. 3-hydroxy-3-methylglutaryl-CoA reductase (HMGCR) protein levels were increased in mutant cells, whereas HMGCR activity was significantly decreased, resulting in reduced cholesterol synthesis. U18666A, an inhibitor of lysosomal cholesterol export, induced cholesterol biosynthetic enzymes; yet, cholesterol synthesis was still reduced. Interestingly, peroxisome deficiency promoted ER-to-Golgi SREBP cleavage-activating protein (SCAP) trafficking even when cells were cholesterol-loaded. Restoration of functional peroxisomes normalized regulation of cholesterol synthesis and SCAP trafficking. These results highlight the importance of functional peroxisomes for maintaining cholesterol homeostasis and efficient cholesterol synthesis.

## Introduction

**FIGURE 1:**
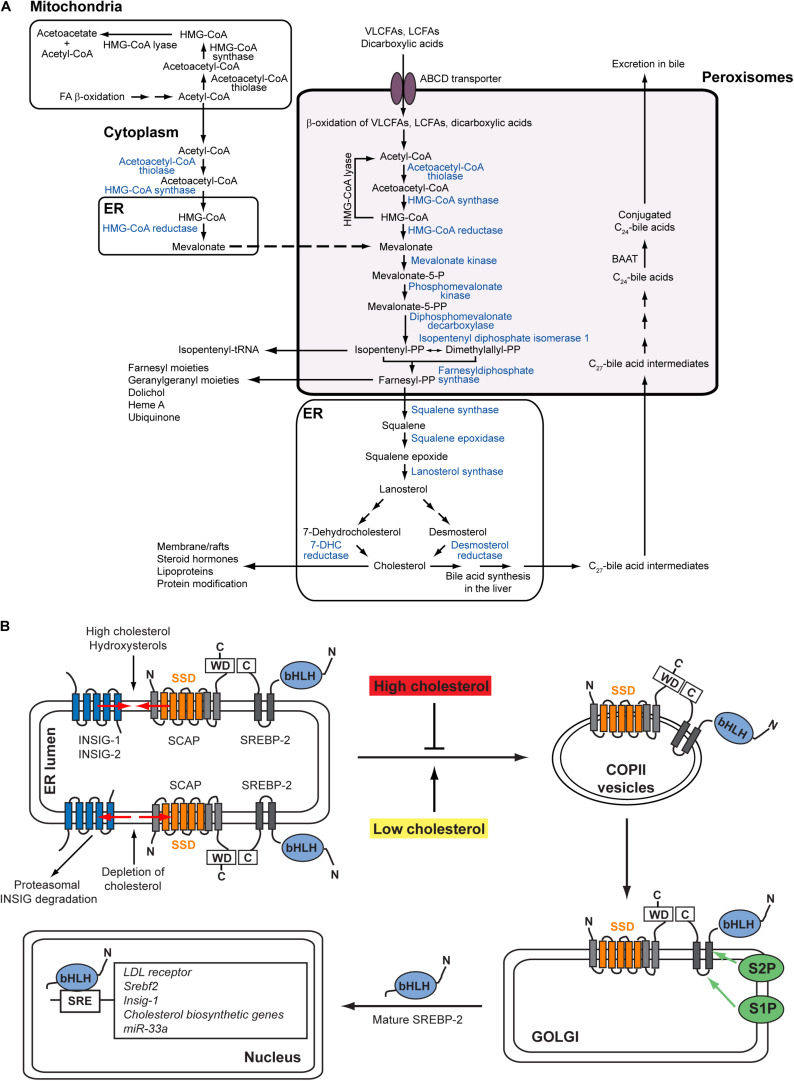
**(A)** Subcellular localization of the cholesterol biosynthetic pathway in mammalian cells. Blue fonts indicate SREBP-2-regulated cholesterol biosynthetic enzymes. With the exception of HMGCR, all enzymes for the conversion of acetyl-CoA to farnesyl diphosphate contain functional peroxisomal targeting signals. Acetyl-CoA derived from peroxisomal β-oxidation of very long-chain fatty acids and dicarboxylic acids is channeled to cholesterol synthesis inside peroxisomes (Weinhofer et al., 2006; Kovacs et al., 2007). Farnesyl diphosphate leaves the peroxisomes and is converted either to cholesterol in the ER or to nonsterol isoprenoids. The conversion of acetyl-CoA to HMG-CoA also occurs in the cytoplasm. The mitochondrial and peroxisomal acetoacetyl-CoA thiolase and the cytosolic and peroxisomal HMG-CoA synthase are encoded by single genes, respectively. In hepatocytes, the conversion of cholesterol to bile acids also partly takes place in peroxisomes. The primary C_24_-bile acids are formed from C_27_-bile acid intermediates by peroxisomal β-oxidation of the side chain, followed by conjugation of the C_24_-bile acids to glycine or taurine by the peroxisomal enzyme BAAT. Figure modified from Figure 1 in Faust and Kovacs (2014). **(B)** Regulation of the SREBP-2 pathway. The precursor SREBP-2 is embedded as inactive transcription factor in ER membranes. In the presence of cholesterol and hydroxysterols (e.g., 25-hydroxycholesterol, 27-hydroxycholesterol), the SCAP-SREBP-2 complex is retained in the ER by interaction with INSIG proteins. Whereas cholesterol binds to SCAP and induces a conformational change in SCAP through which it binds to INSIGs, hydroxysterols bind to INSIGs and cause INSIGs to bind to SCAP. In the absence of sterols, INSIGs dissociate from SCAP, whereupon the INSIGs are ubiquitinated and degraded in proteasomes. The SCAP-SREBP-2 complex is loaded into COPII-coated vesicles and transported to the Golgi. In the Golgi SREBPs are processed sequentially by Site-1 and Site-2 proteases to release the N-terminal transcription factor domain that then enters the nucleus and activates SRE (sterol regulatory element)-containing genes. Figure modified from Figure 2 in Faust and Kovacs (2014).

Cholesterol is an essential lipid constituent of cellular membranes, in particular the plasma membrane, and an obligatory precursor for synthesis of steroid hormones, bile acids, and regulatory oxysterols, as well as a requisite ligand for activation of Hedgehog family proteins. Its synthesis involves more than 20 enzymes distributed over several subcellular compartments. Numerous studies assign the pre-squalene steps of cholesterol biosynthesis to peroxisomes (Figure 1A) [reviewed in Kovacs et al. (2002), Faust and Kovacs (2014)]. With the exception of HMGCR, the enzymes of the pre-squalene segment contain functional peroxisomal targeting signals (PTS) that mediate their import into the peroxisomal matrix (Kovacs et al., 2002; Faust and Kovacs, 2014). We showed that acetyl-CoA derived from peroxisomal β-oxidation of very long-chain fatty acids (i.e., [1,2,3,4-^13^C_4_]docosanoate) and dicarboxylic acids (i.e., [U-^13^C_12_]dodecanedioate) is channeled to cholesterol synthesis inside peroxisomes (Kovacs et al., 2007). The enzymes of the pre-squalene segment of the isoprenoid biosynthetic pathway are also localized to peroxisomes in plants [reviewed in Faust and Kovacs (2014), Henry et al. (2018)]. Furthermore, several enzymes of the isoprenoid biosynthetic pathway are localized within peroxisome-related microbodies called glycosomes in trypanosomatid parasites (Concepcion et al., 1998; Urbina et al., 2002; Colasante et al., 2006; Carrero-Lérida et al., 2009; Güther et al., 2014; Ferreira et al., 2016; Acosta et al., 2019).

Cellular cholesterol homeostasis involves sensing sterol levels in the endoplasmic reticulum (ER) and thereby balancing its uptake, synthesis, efflux, and metabolic conversion to a wide variety of biologically significant products (Russell, 2003; Goldstein et al., 2006; Goldstein and Brown, 2015; Brown et al., 2018). For this cells have established an elaborate feedback system to adjust their sterol content, using both transcriptional and post-transcriptional feedback systems (Wangeline et al., 2017; Brown et al., 2018). The sterol regulatory element-binding protein (SREBP) family of transcription factors is central to this regulatory system (Goldstein et al., 2006). The SREBP family consists of SREBP-1a, SREBP-1c, and SREBP-2 in mammals. SREBP-1a and SREBP-1c are produced from a single gene by use of different promoters and alternative splicing. There is some functional overlap between the different isoforms, but SREBP-1c is mainly responsible for the expression of genes involved in fatty acid, triacylglycerol, and phospholipid biosynthesis, whereas SREBP-2 activates genes involved in the uptake and biosynthesis of cholesterol (Goldstein et al., 2006). SREBP-1a is a potent activator of all SREBP-responsive genes. Mammalian cells supply themselves with cholesterol through receptor-mediated endocytosis of low density lipoproteins (LDL), which carry cholesterol primarily in the form of fatty acid esters (Goldstein and Brown, 2015). After delivery to lysosomes, their cargo of cholesteryl esters is hydrolyzed, releasing free cholesterol, which is then exported from the lysosomes to other membrane-bound organelles and cellular membranes, notably the plasma membrane and the ER (Das et al., 2014; Iaea and Maxfield, 2015). In the ER cholesterol binds to SREBP cleavage-activating protein (SCAP), an ER protein that functions as a sterol sensor and regulates the cleavage and activation of membrane-bound SREBPs (Brown et al., 2018). In sterol-rich cells, SREBPs reside as transcriptionally inactive membrane-bound precursor proteins in the ER by interaction with SCAP and INSIG-1 (insulin-induced gene 1) proteins (Figure 1B). In sterol-depleted cells INSIG-1 dissociates from SCAP, which allows SCAP to escort SREBPs in COPII-coated vesicles from the ER to the Golgi apparatus (Figure 1B). In the Golgi SREBPs are processed sequentially by two proteases (Site-1 and Site-2 protease) to release the N-terminal transcription factor domain that then enters the nucleus and activates SRE (sterol regulatory element)-containing genes (Figure 1B; Horton et al., 2002). After dissociating from SCAP, INSIG-1 is rapidly ubiquitinated and degraded by the proteasome (Goldstein et al., 2006). Whereas cholesterol binds to SCAP and induces a conformational change in SCAP that causes it to bind to INSIG-1, hydroxycholesterols bind to INSIGs and cause INSIGs to bind to SCAP (Brown et al., 2018).

Using the peroxisome-deficient *Pex2* knockout (*Pex2*^–/–^) mouse we have shown the importance of peroxisomes for the maintenance of normal cholesterol homeostasis (Kovacs et al., 2004, 2009, 2012). Total cholesterol levels were similar in the brain, kidney, spleen, heart, and lung of control (*Pex2^+/+^* and *Pex2^+/–^*, hereafter referred to as *Pex2^+/^*) and *Pex2*^–/–^ mice, however, hepatic cholesterol levels in 9-day-old (P9) *Pex2*^–/–^ mice were decreased by 40%, relative to controls, while bile acid (BA) feeding normalized their hepatic cholesterol levels. HMG-CoA reductase (HMGCR), isopentenyl diphosphate isomerase 1 (IDI1), farnesyl diphosphate synthase (FDPS), and squalene synthase (FDFT1) activities and protein levels were significantly increased in the liver of P9-P10 *Pex2*^–/–^ mice, while BA feeding attenuated these enzyme activities and protein levels. Interestingly, the increase in protein levels was significantly greater than the increase in the activities of these enzymes, and the “catalytic efficiency” of HMGCR and IDI1 was significantly decreased in newborn (P0) and untreated as well as BA-fed postnatal *Pex2*^–/–^ mice (Kovacs et al., 2004, 2009, 2012). Notably, a different regulatory pattern of cholesterol enzyme activities was observed in kidneys from *Pex2*^–/–^ *vs*. control mice: the activity of HMGCR was significantly decreased, whereas activities of other cholesterol biosynthetic enzymes (i.e., IDI1, FDPS, FDFT1) were increased (Kovacs et al., 2004, 2009, 2012). Using [^3^H]acetate as substrate, we showed that the rate of cholesterol synthesis in the liver, spleen, lung, and heart of P9 *Pex2*^–/–^ mice was significantly increased compared to controls, which is probably due to the highly increased protein levels and activities of cholesterol biosynthetic enzymes in the tissues of *Pex2*^–/–^ mice (Kovacs et al., 2004, 2009). Interestingly, the rate of cholesterol synthesis was significantly decreased in the brain and kidneys of P9 *Pex2*^–/–^ mice (Kovacs et al., 2004, 2009). The hepatic expression of SREBP-2 target genes was significantly increased both in P0 and postnatal *Pex2*^–/–^ mice. BA feeding reduced the expression of these genes significantly; yet, the mRNA levels were still increased (Kovacs et al., 2004, 2009, 2012). In addition, we showed that peroxisome deficiency activates hepatic ER stress pathways in *Pex2*^–/–^ mice, especially the integrated stress response which is mediated by PKR-like endoplasmic reticulum kinase (PERK) and activating transcription factor-4 (ATF4) signaling; hence, we hypothesized that ER stress leads to dysregulation of the endogenous sterol response mechanism and SREBP-2 pathway induction (Kovacs et al., 2009, 2012; Faust and Kovacs, 2014).

Conflicting reports on the role of peroxisomes in cholesterol biosynthesis have been published from *in vitro* studies using fibroblasts from patients with peroxisomal biogenesis disorders as well as peroxisome-deficient rodent cells [reviewed in Kovacs et al. (2002)]. In summary, activities of peroxisomal cholesterol biosynthetic enzymes were either normal or decreased in fibroblasts of patients with Zellweger spectrum disorders. Three studies employing a total of 24 fibroblast cell lines from patients with disorders of the Zellweger spectrum demonstrated a significantly reduced rate of cholesterol biosynthesis compared to control cells, whereas two studies using a total of seven fibroblast cell lines found that cholesterol biosynthesis rates were similar or higher than those in control fibroblasts. HMGCR activity and the rates of both cholesterol and non-sterol (dolichol) biosynthesis were found to be significantly lower in a study using peroxisome-deficient *Pex2*^–/–^ hamster cells (ZR-78.1C, ZR-82) (Aboushadi and Krisans, 1998), while in another study using ZR-82 cells, both HMGCR activity and the incorporation of [^3^H]acetate into sterols (cholesterol + lanosterol) were increased compared to wild-type CHO-K1 cells (van Heusden et al., 1992). However, *in vitro* studies have not investigated the regulation of cholesterol biosynthesis in peroxisome-deficient cells.

In this study, we investigated the transcriptional regulation of cholesterol biosynthesis in wild-type CHO-K1 cells, three isogenic peroxisome-deficient CHO cell lines (ZR-78.1C, ZR-82, ZR-87) with mutations in the *Pex2* gene, and ZR cells with restored functional peroxisomes after complementation with wild-type *Pex2* cDNA. We also determined the rate of cholesterol biosynthesis as well as activities and protein levels of cholesterol biosynthetic enzymes in these cell lines. Finally, we explored mechanisms that might lead to a dysregulation of the endogenous sterol response in peroxisome-deficient CHO cells.

## Results

### Peroxisome-Deficient CHO Cells

The *Pex2* mutant and peroxisome-deficient CHO cell lines (ZR-78.1C, ZR-82, and ZR-87) were isolated from the CHO-K1 cell line used as control in this study (Zoeller and Raetz, 1986; Zoeller et al., 1989). PEX2 is anchored to the peroxisomal membrane by two membrane-spanning segments, with its N- and C-terminal regions exposed to the cytosol (Harano et al., 1999). The C-terminus of PEX2 contains a RING finger (C_3_HC_4_) motif. The point mutations of *Pex2* in the ZR-78.1C and ZR-82 cell lines have been identified, whereas the mutation of *Pex2* in the ZR-87 cell line is not known (Thieringer and Raetz, 1993). In ZR-78.1C nucleotide G at position 737 was mutated to A, resulting in the conversion of a cysteine residue into a tyrosine residue in the RING finger motif. The mutation in ZR-82 cells introduces a stop codon which leads to the translation of a truncated form of the PEX2 protein. This N-terminal fragment constitutes only one-fifth of the entire protein and lacks both membrane-spanning regions.

**FIGURE 2:**
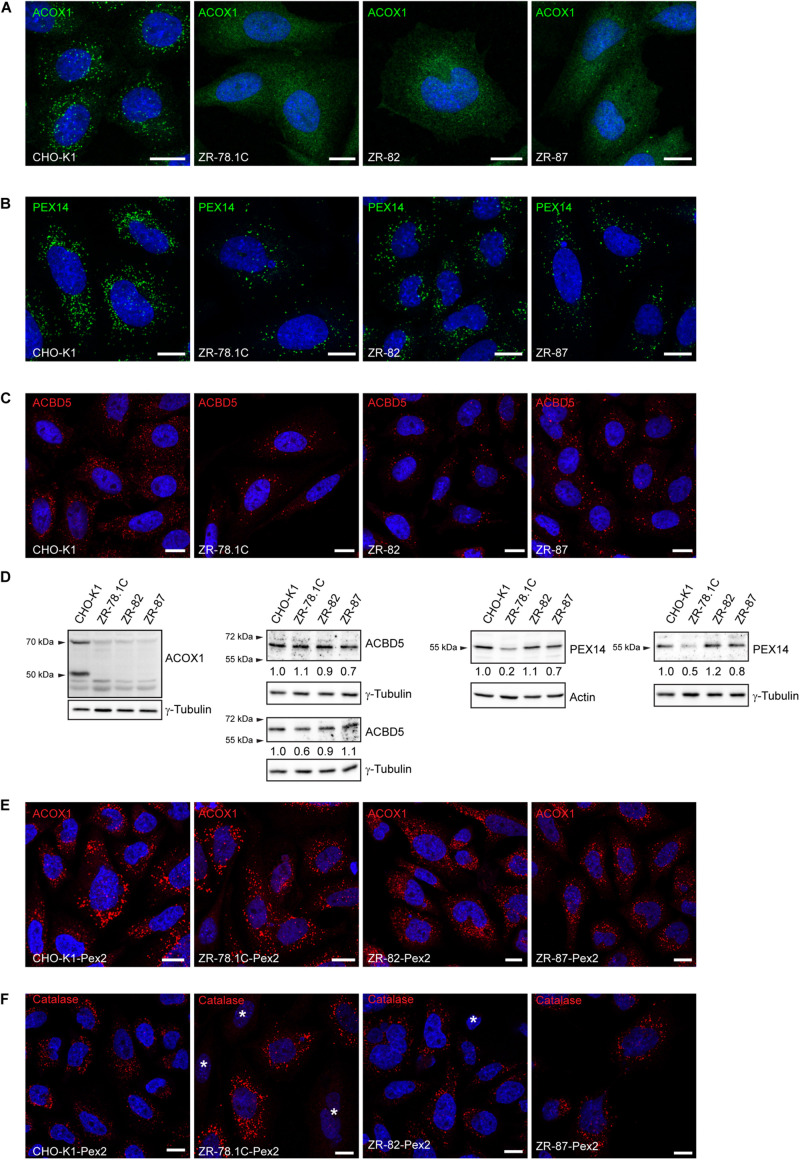
Peroxisome-deficient CHO cells contain peroxisome membraneghosts. **(A)** Peroxisomes were detected with an antibody against the peroxisomal matrix protein ACOX1. The nuclei were stained with DAPI (blue). Note the cytoplasmic localization of Acox1 in the peroxisome-deficient ZR-78.1C, ZR-82, and ZR-87 cells. **(B)** Peroxisomes were detected with an antibody against the peroxisomal membrane protein PEX14. Note the presence of peroxisome membrane ghosts in peroxisome-deficient CHO cells. The number of peroxisome membrane ghosts in peroxisome-deficient CHO cells is significantly lower than the number of peroxisomes in CHO-K1 cells. **(C)** Peroxisomes were visualized with an antibody against the peroxisomal tail-anchored protein ACBD5. Note that ACBD5 localizes to peroxisome membrane ghosts in peroxisome-deficient CHO cells. **(D)** Immunoblots of total cell lysates with antibodies against peroxisomal matrix and membrane proteins. Numbers at the bottom of the blots indicate the fold change in protein levels in peroxisome-deficient cells relative to that in CHO-K1 cells, which were arbitrarily defined as 1. **(E,F)** Functional peroxisomes are restored in peroxisome-deficient CHO mutants (ZR-78.1C, ZR-82, ZR-87) upon complementation with rat *Pex2* cDNA. Cells were immunostained with antibodies against the peroxisomal matrix proteins ACOX1 **(E)** and catalase **(F)**. The nuclei were stained with DAPI (blue). An asterisk indicates non-transfected cells without import-competent peroxisomes. The scale bars represent 10 μm.

To assess if a complete or partial absence of peroxisomes exist in our cell lines, an immunofluorescence analysis was performed. The immunofluorescence pattern obtained for acyl-CoA oxidase 1 (ACOX1), a peroxisomal matrix protein involved in peroxisomal fatty acid β-oxidation, showed the characteristic punctuate peroxisomal distribution in CHO-K1 cells (Figure 2A) and a diffuse, cytoplasmic fluorescence in peroxisome-deficient CHO cells (Figure 2A), consistent with mislocalization of ACOX1 to the cytoplasm. A punctuate peroxisomal staining pattern for the peroxisomal membrane proteins PEX14 and ACBD5 was observed in CHO-K1 cells (Figures 2B,C). In peroxisome-deficient CHO cells, PEX14 and ACBD5 were present in less abundant cellular vesicles, consistent with peroxisome membrane ghosts (Figures 2B,C). These findings are consistent with the established function of PEX2 in the import of peroxisomal matrix proteins, but not peroxisomal membrane proteins.

Acyl-CoA oxidase 1 is synthesized as a precursor protein of 72 kDa and undergoes proteolytic cleavage after import into peroxisomes, resulting in subunits of 52 and 20.5 kDa (Miyazawa et al., 1987). In peroxisome-deficient CHO cells only a slight band of the uncleaved precursor protein was seen (Figure 2D), consistent with lack of peroxisomal protein import and rapid degradation outside of peroxisomes (Schram et al., 1986; Zoeller et al., 1989). Interestingly, the protein levels of ACBD5 were not significantly altered in peroxisome-deficient cells (Figure 2D), whereas PEX14 levels were decreased in ZR-78.1C and ZR-87 cells (Figure 2D).

In order to restore functional peroxisomes, we transfected the peroxisome-deficient CHO cells with wild-type rat *Pex2* cDNA and stably selected transfected cells with G418. Transfection with *Pex2* restored peroxisomal matrix protein import, since a normal punctate fluorescence pattern was observed with ACOX1 and catalase staining (Figures 2E,F; *cf*. also Supplementary Figures S1A,B). The peroxisomes of the *Pex2*-transfected mutant cell lines were morphologically indistinguishable from peroxisomes found in the wild-type CHO-K1 and *Pex2*-transfected CHO-K1 cells. Hereafter we refer to these transfected cells as ZR-78.1C-Pex2, ZR-82-Pex2, and ZR-87-Pex2.

### Transcriptional Activity of Cholesterogenic Promoter-Reporters Is Increased in Peroxisome-Deficient CHO Cells

**FIGURE 3:**
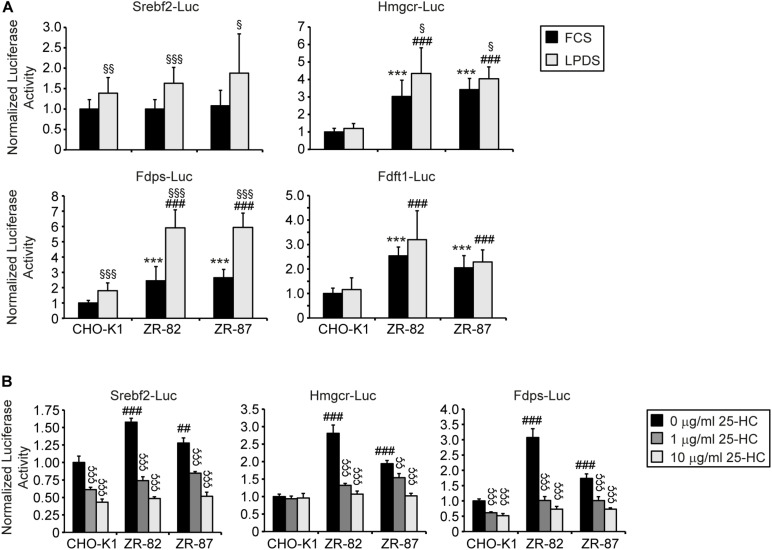
Promoter activities of *Srebf2* and the cholesterol biosynthetic genes *Hmgcr*, *Fdps*, and *Fdft1* in CHO-K1 and peroxisome-deficient CHO cells. **(A)** The indicated plasmids were transfected into cells as described in *Materials and Methods.* 24 hours after transfection, the medium was switched to medium containing either 10% FCS or 5% LPDS and incubated for 24 h, after which the cells were harvested and assayed for dual luciferase activities as described in section “Materials and Methods.” **(B)** The indicated plasmids were transfected into cells and 24 hours after transfection the medium was switched to medium supplemented with 5% LPDS and 0, 1, or 10 μg/ml 25-hydroxycholesterol (25-HC). After 24 h incubation, cells were harvested and assayed for dual luciferase activities. Data are presented as firefly luciferase activity normalized to *Renilla* luciferase activity and each value represents the mean ± SD of three independent transfection experiments done in quadruplicate. Statistical analysis was performed using Student’s *t*-test or Student’s t-test with Welch’s correction or one-way ANOVA followed by Dunnett’s multiple comparisons test. *** *p* < 0.001 vs. FCS-cultured CHO-K1. ^##^*p* < 0.01; ^###^*p* < 0.001 vs. LPDS-cultured CHO-K1. ^§^ *p* < 0.05; ^§§^ *p* < 0.01; ^§§§^ *p* < 0.001 vs. corresponding FCS-cultured cell line. ^ÇÇ^ *p* < 0.01; ^ÇÇÇ^ *p* < 0.001 vs. corresponding cell line incubated in medium containing 5% LPDS without 25-HC.

In order to determine whether sterol-regulated transcription of cholesterogenic genes is affected in peroxisome-deficient CHO cells, luciferase reporter constructs containing the sterol response element (SRE) of SREBP-2 (*Srebf2*), HMG-CoA reductase (*Hmgcr*), farnesyl diphosphate synthase (*Fdps*), or squalene synthase (*Fdft1*) were transiently transfected into wild-type CHO-KI and peroxisome-deficient ZR-82 and ZR-87 cells. For this cells were incubated in medium supplemented with either 10% FCS or 5% lipoprotein-deficient serum (LPDS), the latter a condition known to upregulate the SREBP-2 pathway. Expression of the *Srebf2* and *Fdps* luciferase reporter genes was increased significantly in all cell lines when incubated in medium with LPDS (Figure 3A), indicating the responsiveness of SREBP to low cholesterol levels. However, *Fdps* luciferase activity was higher in peroxisome-deficient cells compared with CHO-K1 cells. It is interesting to note that the expression of the *Hmgcr*, *Fdps*, and *Fdft1* luciferase reporter constructs in peroxisome-deficient cells was significantly increased compared with CHO-K1 even when the cells were incubated in medium containing 10% FCS and therefore sufficient amount of extracellular cholesterol was available (Figure 3A). The expression of the *Hmgcr* luciferase reporter constructs was only slightly enhanced further in peroxisome-deficient cells incubated in medium with LPDS compared to FCS-cultured cells, whereas the expression was not increased at all in LPDS-cultured CHO-K1 cells. It has been shown that LDL in FCS provides enough cholesterol to suppress SREBP-2 cleavage in wild-type CHO and HEK-293 cells (Hannah et al., 2001; Lee and Ye, 2004). Whereas the expression of the *Fdft1* luciferase reporter gene was increased in FCS-cultured peroxisome-deficient cells compared with CHO-K1, culturing all cell lines in medium with 5% LPDS did not increase the expression of the *Fdft1* luciferase reporter (Figure 3A).

We then tested whether cholesterogenic promoter activity in peroxisome-deficient cells can be suppressed by incubation with 5% LPDS supplemented with 1 and 10 μg/ml 25-hydroxycholesterol (25-HC) (Figure 3B). Unlike LDL-borne cholesterol, 25-HC added to the culture medium in solvent enters the cell and reaches the ER without first traversing lysosomes (Abi-Mosleh et al., 2009). 25-HC suppressed *Srebf2*, *Hmgcr*, and *Fdps* promoter activity both in CHO-K1 and peroxisome-deficient ZR-82 and ZR-87 cells (Figure 3B) indicating the responsiveness of SREBP to hydroxycholesterols. Taken together, these results show that peroxisome deficiency activates expression of cholesterogenic promoter-reporter genes, even though total cholesterol levels are unchanged when compared to wild-type CHO-K1 cells (Table 1).

**TABLE 1:** Cholesterol levels in CHO-K1 and peroxisome-deficient CHO cells.

		**Cholesterol [ng/μg protein]**	
**Cell line**	**24 h 10% FCS**	**24 h 5% LPDS**	**48 h 5% LPDS**
CHO-K1	54.1 ± 0.8	19.7 ± 0.4	28.2 ± 2.3
ZR-82	53.4 ± 1.5	25.5 ± 0.4	29.0 ± 5.0
ZR-87	58.0 ± 0.8	26.7 ± 0.8	25.9 ± 0.4

*Each value represents the average ± range (*n* = 2).*

### Expression of Cholesterol Biosynthetic Genes Is Increased in Peroxisome-Deficient CHO Cells

**FIGURE 4:**
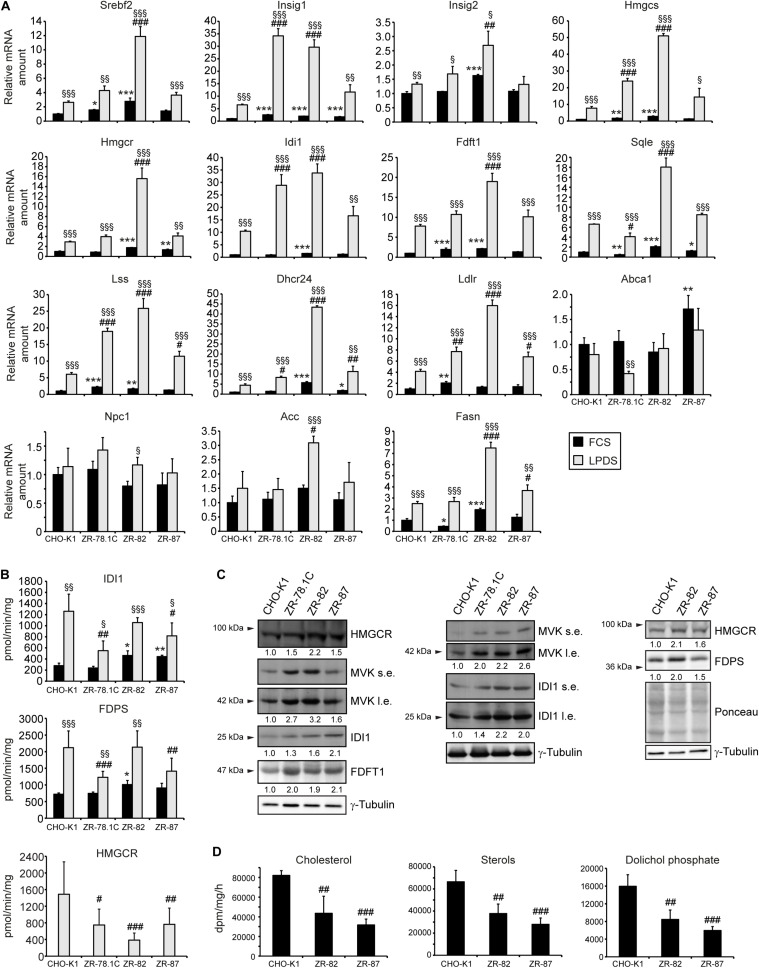
**(A)** Expression of genes involved in cholesterol biosynthesis and its regulation, cholesterol efflux, and fatty acid synthesis in CHO-K1 and peroxisome-deficient CHO cells cultured in medium containing 10% FCS or 5% LPDS for 24 h. Each value represents the amount of mRNA relative to that in FCS-cultured CHO-K1, which was arbitrarily defined as 1. Data are mean ± SD (*n* = 3). **(B)** Activities of the cholesterol biosynthetic enzymes HMGCR, IDI1, and FDPS. Each value represents the average of 10-17 experiments, performed in duplicate. Data are mean ± SD. **(C)** Immunoblot analysis of cholesterol biosynthetic enzymes HMGCR, MVK, IDI1, FDPS, and squalene synthase (FDFT1) in cells after incubation in medium containing 5% LPDS for 24 h. Numbers at the bottom of the blots indicate the fold change in protein levels in peroxisome-deficient cells relative to that in CHO-K1 cells, which were arbitrarily defined as 1. l.e., long exposure; s.e., short exposure. **(D)** Rate of biosynthesis of cholesterol, total sterols, and dolichols in CHO-K1 and peroxisome-deficient ZR-82 and ZR-87 cells as measured by the incorporation of [^14^C]acetate. Data are mean ± SD of three experiments, performed in duplicate. Statistical analysis was performed using Student’s t-test or Student’s t-test with Welch’s correction or one-way ANOVA followed by Dunnett’s multiple comparisons test. *, *p* < 0.05; **, *p* < 0.01; ***, *p* < 0.001 vs. FCS-cultured CHO-K1. ^#^*p* < 0.05; ^##^*p* < 0.01; ^###^*p* < 0.001 vs. LPDS-cultured CHO-K1. ^§^ *p* < 0.05; ^§§^ *p* < 0.01; ^§§§^ *p* < 0.001 vs. corresponding FCS-cultured cell line.

Next, we determined the mRNA expression of genes involved in cholesterol biosynthesis and their regulation, in cholesterol efflux, and in fatty acid synthesis in CHO-K1 and peroxisome-deficient CHO cells cultured in medium containing 10% FCS or 5% LPDS for 24 h. Again, the expression of *Srebf2*, *Insig1*, cholesterol biosynthetic genes (*Hmgcs*, *Hmgcr*, *Idi1*, *Fdft1*, *Sqle*, *Lss*, *Dhcr24*), *Ldlr*, and *Fasn* was significantly increased in all cell lines when incubated in medium containing LPDS (Figure 4A). However, the expression of these genes was much more strongly induced in peroxisome-deficient cells, especially in ZR-82 cells, than in CHO-K1 cells. Importantly, the expression of cholesterol biosynthetic genes and its regulators *Srebf2* and *Insig1* also was significantly increased in peroxisome-deficient cells compared with CHO-K1 when the cells were incubated in medium containing 10% FCS (Figure 4A; *cf*. also Supplementary Figure S2). These data corroborate the promoter studies shown in Figure 3. Next, exit of cholesterol from lysosomes requires the membrane-bound Niemann-Pick C1 (NPC1) protein (Kwon et al., 2009); however, we did not detect any changes in *Npc1* expression at least in peroxisome-deficient cells (Figure 4A). Hence, although it is well-known that all cholesterol biosynthetic genes are regulated by SREBP-2 (Horton et al., 2002), the magnitude of response in their expression varied significantly among the genes tested, indicating that their levels do not depend on the expression level of SREBP-2 alone.

### Activities of Cholesterol Biosynthetic Enzymes and Protein Levels Are Altered in Peroxisome-Deficient CHO Cells

To examine the effect of peroxisome deficiency on the activities of cholesterol biosynthetic enzymes, we measured the activities of HMGCR, IDI1, and FDPS in CHO-K1 and peroxisome-deficient cells (Figure 4B). HMGCR, which catalyzes the rate-limiting step of sterol biosynthesis, is localized mainly in the ER, whereas IDI1 and FDPS are localized predominantly in peroxisomes (Kovacs et al., 2002; Faust and Kovacs, 2014). HMGCR activity was decreased 50% in ZR-78.1C and ZR-87 cells and 75% in ZR-82 cells, corroborating previously published data (Aboushadi and Krisans, 1998). The activity of IDI1 and FDPS was similar or increased in FCS-cultured peroxisome-deficient cells compared with CHO-K1 cells. IDI1 and FDPS activities were significantly increased in all cell lines when incubated in medium containing LPDS, however, the activities were somewhat lower in peroxisome-deficient cells compared with CHO-K1 cells.

Western blot analysis of proteins involved in cholesterol biosynthesis was performed to determine whether the measured activities are a reflection of the protein levels. The protein levels of HMGCR, mevalonate kinase (MVK), IDI1, FDPS, and FDFT1 were significantly increased in peroxisome-deficient cells compared with CHO-K1 (Figure 4C). Thus, decreased HMGCR and IDI1 activities in the peroxisomal-deficient cells are not a reflection of their protein levels, however, gene expression data gave reason to expect even higher protein levels of cholesterol biosynthetic genes in peroxisome-deficient cells.

### Rates of Cholesterol and Dolichol Biosynthesis Are Decreased in Peroxisome-Deficient Cells

To evaluate the effects of the decrease in HMGCR, IDI1, and FDPS activities in peroxisome-deficient cells, we measured the rate of sterol (cholesterol) and non-sterol (dolichols) biosynthesis in CHO-K1, ZR-82, and ZR-87 cells after incubating the cells with [^14^C]acetate. The rates of cholesterol and dolichol biosynthesis were significantly reduced in the peroxisomal-deficient cells as compared to the CHO-K1 cells (Figure 4D and Supplementary Table S1). These data corroborate a previously published study showing that cholesterol and dolichol biosynthesis rates were decreased in ZR-78.1C and ZR-82 cells when incubated with either [^3^H]acetate or [^3^H]mevalonate (Aboushadi and Krisans, 1998). However, despite decreased biosynthesis rates, total cholesterol levels were similar in CHO-K1 and peroxisome-deficient cells cultured in medium containing either 10% FCS or 5% LPDS for 24 and 48 h (Table 1).

### U18666A Treatment Activates the SREBP-2 Pathway in CHO-K1 and Peroxisome-Deficient CHO Cells

**FIGURE 5:**
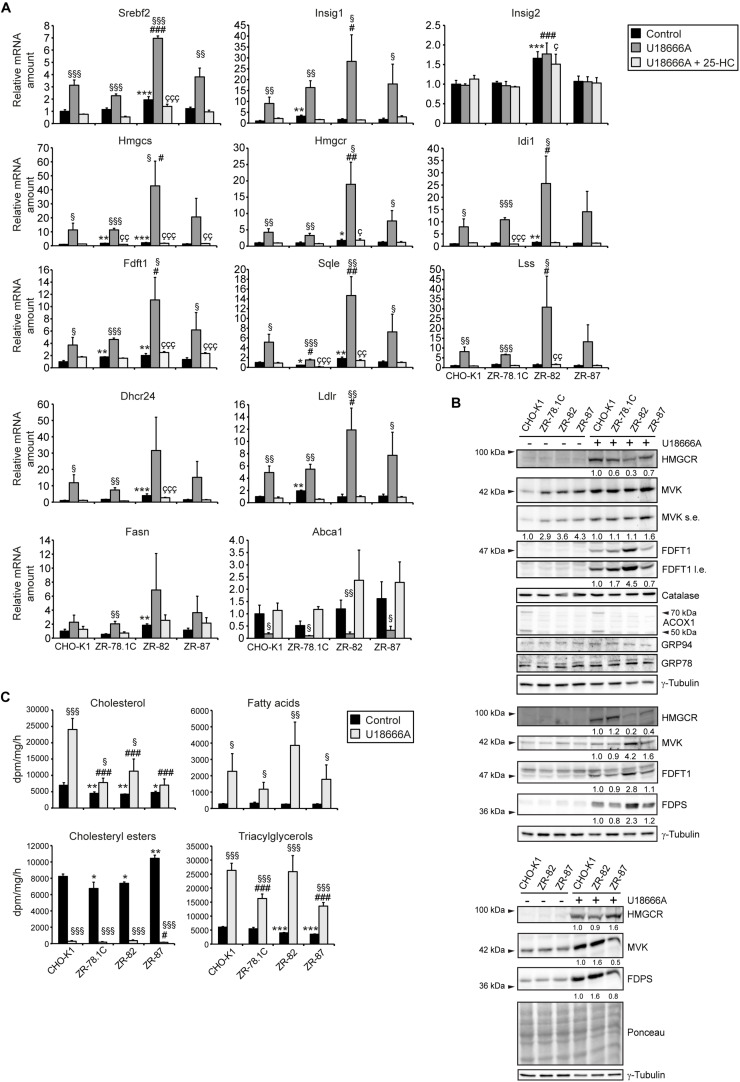
**(A)** Expression of genes involved in cholesterol biosynthesis and its regulation, cholesterol efflux, and fatty acid synthesis in CHO-K1 and peroxisome-deficient CHO cells cultured in medium containing 10% FCS and 0.1% EtOH as solvent control or 10 μM U18666A or 10 μM U18666A and 10 μg/ml 25-hydroxycholesterol (25-HC) for 24 h. Each value represents the amount of mRNA relative to that in control CHO-K1, which was arbitrarily defined as 1. Data are mean ± SD (*n* = 3). **(B)** Immunoblots of total cell lysates with antibodies against cholesterol biosynthetic enzymes (HMGCR, MVK, FDPS, and FDFT1), peroxisomal (catalase, ACOX1) and ER (GRP78, GRP94) proteins after treatment with 10 μM U18666A for 24 h. Shown are immunoblots from three experiments. **(C)** Rate of biosynthesis of cholesterol, cholesteryl esters, fatty acids, and triacylglycerols in vehicle- and U18666A-treated CHO-K1 and peroxisome-deficient ZR-78.1C, ZR-82, and ZR-87 cells as measured by the incorporation of [^14^C]acetate. Lipids were separated by thin-layer chromatography after [^14^C]acetate labeling. Data are mean ± SD (*n* = 3–6). Statistical analysis was performed using Student’s *t*-test or Student’s *t*-test with Welch’s correction or one-way ANOVA followed by Dunnett’s multiple comparisons test. *p < 0.05; **p < 0.01; ***p < 0.001 vs. vehicle (EtOH)-treated CHO-K1. ^#^*p* < 0.05; ^##^*p* < 0.01; ^###^*p* < 0.001 vs. U18666A-treated CHO-K1. ^Ç^*p* < 0.05; ^ÇÇ^ *p* < 0.01; ^ÇÇÇ^ *p* < 0.001 vs. U18666A- and 25-HC-treated CHO-K1. ^§^ *p* < 0.05; ^§§^ *p* < 0.01; ^§§§^ *p* < 0.001 vs. corresponding vehicle-treated cell line.

A recent study suggested that peroxisomes play a critical role in the transport of LDL-derived cholesterol from the lysosome to the plasma membrane and other intracellular compartments via a lysosome-peroxisome membrane contact site (Chu et al., 2015). Knockdown of *ABCD1*, *PEX1*, and *PEX26* in HeLa cells using shRNAs led to accumulation of cholesterol in lysosomes to a similar extent as in cells from Niemann-Pick disease type C (NPC) patients. Therefore, we aimed to determine the effect of U18666A on the SREBP-2 pathway in CHO-K1 and peroxisome-deficient cells. The amphiphilic compound U18666A has been shown to bind to Niemann-Pick C1 (NPC1) and inhibit cholesterol export from lysosomes (Liscum and Faust, 1989; Liscum, 1990; Lu et al., 2016), leading to SREBP-2 activation (Colgan et al., 2007). If peroxisome deficiency leads to an accumulation of cholesterol in lysosomes (Chu et al., 2015), U18666A should activate the SREBP-2 pathway only in CHO-K1 cells, but should not lead to further activation in ZR-78.1C, ZR-82, and ZR-87 cells. The expression of SREBP-2 target genes was determined in cells that were grown in cholesterol-containing medium (10% FCS) supplemented with 10 μM U18666A for 24 h. U18666A significantly increased the mRNA levels of *Srebf2*, *Insig1*, and cholesterol biosynthetic genes both in CHO-K1 and peroxisome-deficient cells compared with vehicle-treated cells (Figure 5A). The increase was similar in CHO-K1, ZR-78.1C, and ZR-87 cells, whereas ZR-82 cells again showed a much stronger increase, even though with a larger standard deviation. U18666A treatment significantly decreased the expression of the cholesterol efflux transporter *Abca1* (ATP-binding cassette transporter A1) in all cell lines (Figure 5A). The addition of 10 μg/ml 25-HC significantly suppressed the activation of SREBP-2 target genes in U18666A-treated cells (Figure 5A), indicating that the sensitivity towards 25-HC is functional in peroxisome-deficient cells.

U18666A treatment increased the protein levels of the cholesterol biosynthetic enzymes MVK, FDPS, and FDFT1 in all cell lines compared with vehicle-treated cells (Figure 5B). Interestingly, despite highly elevated mRNA levels, HMGCR protein levels in U18666A-treated ZR-82 cells were lower compared with CHO-K1, ZR-78.1C, and ZR-87 cells, whereas the FDPS and FDFT1 protein levels were higher in ZR-82 cells. The protein levels of catalase and the ER proteins GRP78 and GRP94 were similar in CHO-K1 and peroxisome-deficient cells.

To assess levels of *de novo* lipogenesis upon U18666A treatment we measured total *de novo* lipid production by quantifying incorporation of radioactively labeled [^14^C]acetate into lipids. Cholesterol synthesis was significantly decreased in vehicle-treated peroxisome-deficient cells compared with CHO-K1 cells (Figure 5C). U18666A treatment significantly increased the rate of cholesterol biosynthesis in all cell lines, however, cholesterol biosynthesis was significantly lower in peroxisome-deficient cells compared with CHO-K1 cells (Figure 5C). U18666A treatment significantly decreased as expected the rate of cholesteryl ester synthesis in all cell lines, whereas the rates of fatty acid and triacylglycerol synthesis were increased (Figure 5C). Interestingly, the rate of cholesteryl ester synthesis was similar in all cell lines cultivated in FCS, suggesting that they have a similar supply of cholesterol in the ER. In summary, U18666A induced mRNA and protein levels of cholesterol biosynthetic enzymes in peroxisome-deficient cells, however, again the rates of cholesterol synthesis were significantly lower than in CHO-K1 cells.

### Expression of SREBP-2 Target Genes in Extrahepatic Tissues of SW/129 *Pex2*^–/–^ Mice

**FIGURE 6:**
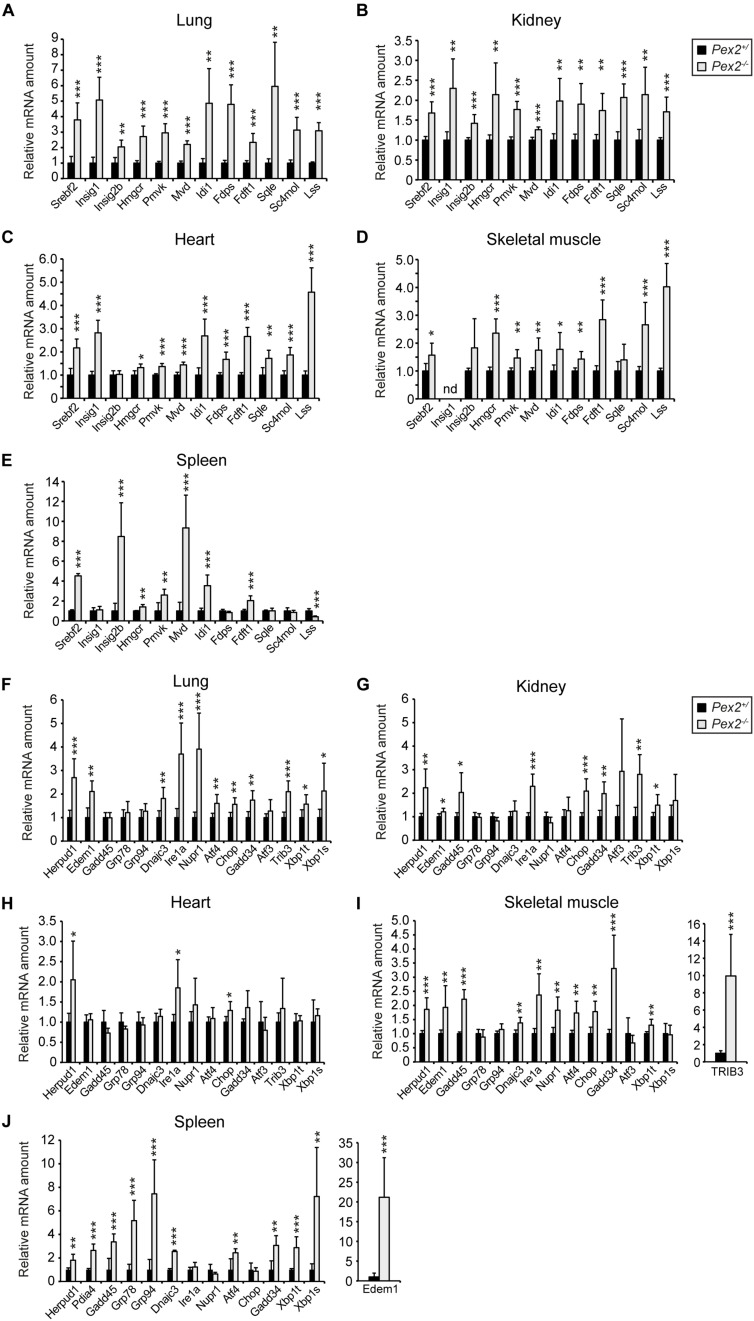
Expression of SREBP-2 regulated genes in lung **(A)**, kidney **(B)**, heart **(C)**, skeletal muscle **(D)**, and spleen **(E)** from P10 SW/129 control and *Pex2*^–/–^ mice. **(F–J)** Expression of UPR target genes in lung **(F)**, kidney **(G)**, skeletal muscle **(H)**, heart **(I)**, and spleen **(J)** from P10 SW/129 control and *Pex2*^–/–^ mice. Each value represents the amount of mRNA relative to that in control mice, which was arbitrarily defined as 1. Data are mean ± SD (*n* = 6 for control and *Pex2*^–/–^ mice). Statistical analysis was performed using Student’s *t*-test or Student’s *t*-test with Welch’s correction. *p < 0.05; **p < 0.01; ***p < 0.001 vs. control mice. nd, not detected.

We reported previously that total cholesterol levels were similar in the lung, kidney, heart, and spleen of P10 SW/129 *Pex2*^–/–^ mice when compared with control mice, whereas the rate of cholesterol synthesis was significantly increased in the lung, heart, and spleen and decreased in the kidney (Kovacs et al., 2004, 2009). As total cholesterol levels were similar in P10 SW/129 *Pex2*^–/–^ and control mice, we examined the expression of *Srebf2*, *Insig1*, *Insig2*, and cholesterol biosynthetic genes (*Hmgcr*, *Pmvk*, *Mvd*, *Idi1*, *Fdps*, *Fdft1*, *Sqle*, *Sc4mol*, *Lss*) in these tissues as well as skeletal muscle. Indeed, the mRNA levels of all these genes were significantly increased in *Pex2*^–/–^ mice (Figures 6A–E). In summary, there is a very similar mRNA up-regulation of *Srebf2* and its target genes in extrahepatic tissues compared with livers of P0 and postnatal *Pex2*^–/–^ mice (Kovacs et al., 2004, 2009, 2012). Taken together, these data corroborate our findings in CHO cell lines, which indicate that functional peroxisomes are necessary for efficient cholesterol biosynthesis.

### ER Stress Does Not Activate the SREBP-2 Pathway in CHO-K1 Cells and Peroxisome-Deficient CHO Cells

**FIGURE 7:**
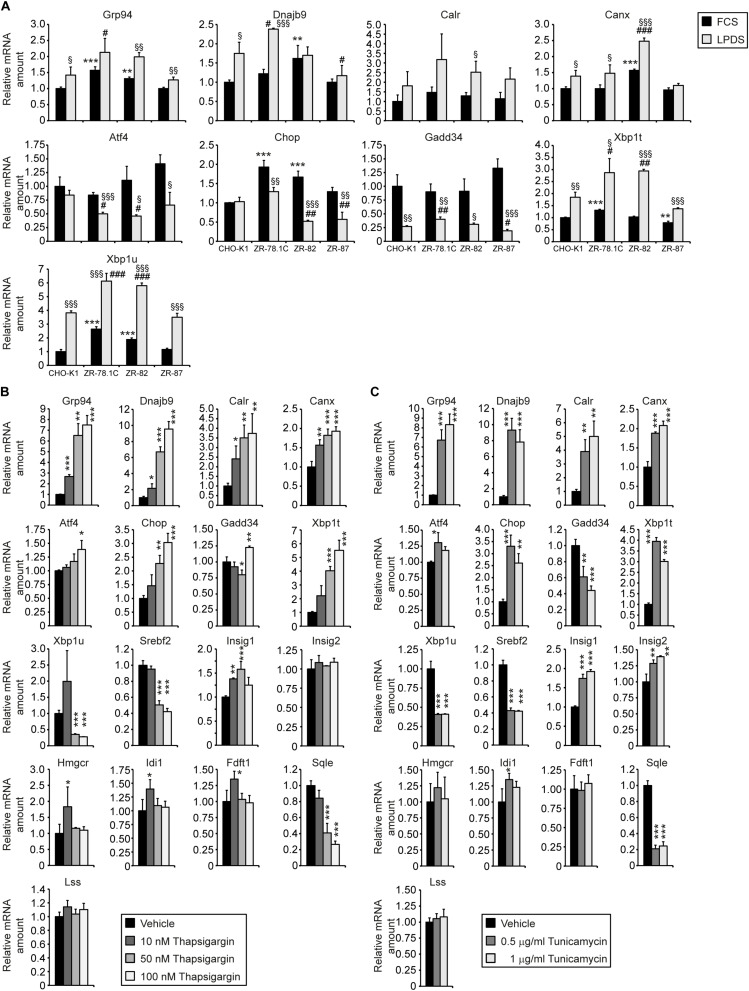
ER stress does not induce the SREBP-2 pathway in CHO-K1 cells. **(A)** Expression of UPR target genes in CHO-K1 and peroxisome-deficient CHO cells cultured in medium containing 10% FCS or 5% LPDS for 24 h. Each value represents the amount of mRNA relative to that in FCS-cultured CHO-K1, which was arbitrarily defined as 1. Data are mean ± SD (*n* = 3). *p < 0.05; **p < 0.01; ***p < 0.001 vs. FCS-cultured CHO-K1. ^#^*p* < 0.05; ^##^*p* < 0.01; ^###^*p* < 0.001 vs. LPDS-cultured CHO-K1. ^§^ *p* < 0.05; ^§§^ *p* < 0.01; ^§§§^ *p* < 0.001 vs. corresponding FCS-treated cell line. **(B,C)** CHO-K1 cells were cultured in medium containing 10% FCS and DMSO as solvent control or various concentrations of thapsigargin **(B)** and tunicamycin **(C)** for 18 h. Each value represents the amount of mRNA relative to that in control CHO-K1, which was arbitrarily defined as 1. Data are mean ± SD (*n* = 3). *p < 0.05; **p < 0.01; ***p < 0.001 vs. solvent (DMSO)-treated CHO-K1. Statistical analysis was performed using Student’s *t*-test or Student’s *t*-test with Welch’s correction or one-way ANOVA followed by Dunnett’s multiple comparisons test.

We showed that peroxisome deficiency activates hepatic ER stress pathways, especially the integrated stress response mediated by PERK and ATF4 signaling, in livers of newborn and postnatal *Pex2*^–/–^ mice (Kovacs et al., 2009, 2012). Studies have shown that ER stress induces SREBP-2 activation independently of intracellular cholesterol concentration in various cell lines (Werstuck et al., 2001; Lee and Ye, 2004; Colgan et al., 2007; Lhoták et al., 2012). The analysis of the mRNA expression of several unfolded protein response (UPR) target genes in extrahepatic tissues of P10 *Pex2*^–/–^ mice showed that ER stress is also present in the lung, kidney, skeletal muscle, heart, and spleen (Figures 6F–J). To determine whether ER stress is also present in peroxisome-deficient CHO cells, we examined the mRNA expression of several UPR target genes in cells cultured in medium containing 10% FCS or 5% LPDS for 24 h (Figure 7A). The expression of most UPR target genes was, with the exception of *Chop* (C/EBP homologous protein), somewhat similar in CHO-K1 and peroxisome-deficient cells cultured in FCS- or LPDS-containing medium. *Chop* was significantly increased in all peroxisome-deficient cells compared with CHO-K1 when grown in medium containing 10% FCS. Culturing cell in 5% LPDS increased the expression of *Xbp1t* (*total*) and *Xbp1u* (*unspliced*) in all cell lines, however, the expression levels of the active transcription factor *Xbp1s* (*spliced*) were very low (C_*T*_ > 38) and could not be analyzed. Interestingly, *Xbp1t* and *Xbp1u* mRNA levels were increased in peroxisome-deficient CHO cells, but the expression of *Xbp1s* was again too low to be evaluated.

Next, we investigated whether induction of ER stress activates the SREBP-2 pathway in CHO-K1 cells. CHO-K1 cells were treated with various concentrations of the ER stress inducers thapsigargin (Tg), an inhibitor of the ER Ca^2+^-dependent ATPase, or tunicamycin (Tm), an inhibitor of protein N-glycosylation (the latter resulting in misfolding of proteins that normally would be glycosylated). Quantitative real-time PCR analysis revealed that Tg and Tm induced a significant dose-dependent increase in mRNA levels of various ER stress markers (*Grp94*, *Dnajb9*, *Calr*, *Canx*, *Atf4*, *Chop*, *Gadd34*, *Xbp1t*), however, the expression of SREBP-2 target genes was in general not induced in Tg- or Tm-treated CHO-K1 cells (Figures 7B,C). The mRNA levels of *Srebf2* and *Sqle* were decreased in Tg- and Tm-treated cells, and only *Insig1* expression was increased.

In summary, the increased expression of cholesterol biosynthetic genes in peroxisome-deficient CHO cells cannot be ascribed to ER stress, and activation of ER stress in CHO-K1 cells does not lead to an activation of the SREBP-2 pathway.

### mTORC1 and MAPK Pathways Do Not Activate the SREBP-2 Pathway in Peroxisome-Deficient CHO Cell

**FIGURE 8:**
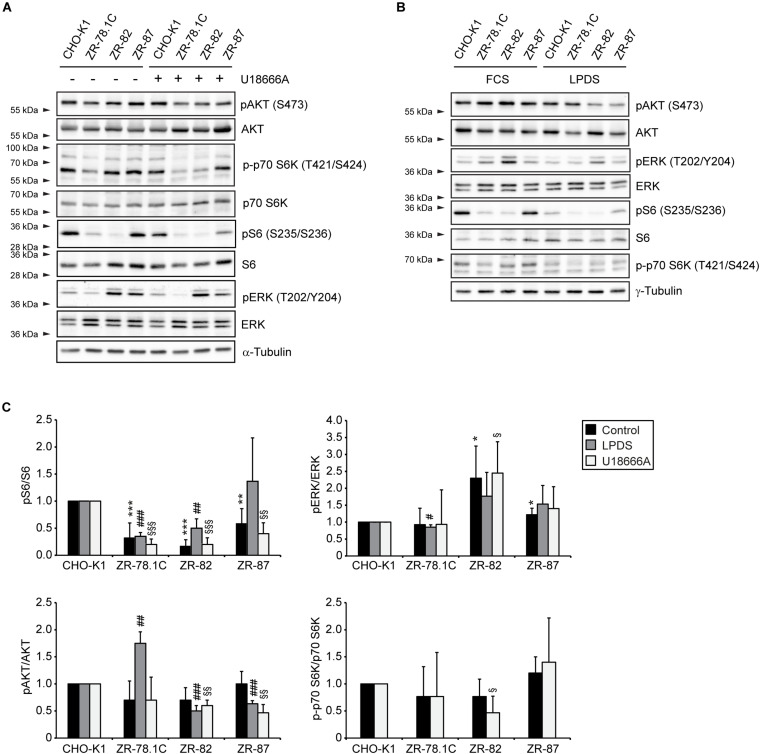
mTORC1, AKT, and MAPK signaling in peroxisome-deficient CHO cells. **(A,B)** Immunoblot analysis of the mTORC1, AKT, and MAPK pathways of total cell lysates after treatment with 10 μM U18666A **(A)** or incubation in medium supplemented with 5% LPDS **(B)** for 24 h. Western blots from a representative of 2–3 independent experiments are shown in **panels A,B**. **(C)** For quantification, pAKT, pS6, pERK, and p-p70 S6K levels were normalized to the corresponding total AKT, S6, ERK, and p70 S6K levels, respectively. Protein ratios are expressed relative to that in CHO-K1 cells, which were arbitrarily defined as 1 for each treatment. Data are mean ± SD (*n* = 2–3). Statistical analysis was performed using Student’s *t*-test or Student’s *t*-test with Welch’s correction. ***p < 0.001 vs. FCS-cultured CHO-K1. ^#^*p* < 0.05; ^##^*p* < 0.01; ^###^*p* < 0.001 vs. LPDS-cultured CHO-K1. ^§^ *p* < 0.05; ^§§^ *p* < 0.01; ^§§§^ *p* < 0.001 vs. U18666A-treated CHO-K1.

Next, we investigated whether activated mTORC1 (mechanistic target of rapamycin complex 1) triggers activation of the SREBP-2 pathway in peroxisome-deficient cells. mTORC1 has been shown to activate SREBP-2 in mammalian cells through several mechanisms, including, at least in some cell types, through p70 ribosomal S6 kinase (p70 S6K) (Peterson et al., 2011; Wang et al., 2011; Eid et al., 2017). The only kinase known to be activated directly by mTORC1 is p70 S6K, which subsequently activates ribosomal S6 protein (S6) (Laplante and Sabatini, 2012). We assessed mTORC1 activity by analyzing the phosphorylation of S6 and p70 S6K in lysates from CHO-K1 and peroxisome-deficient cells treated with U18666A or cultured in the presence of FCS or LPDS for 24 h (Figures 8A–C). Phosphorylation of S6 on Ser235/236 was decreased in peroxisome-deficient cells compared with CHO-K1 in all three culture conditions. Phosphorylation of p70 S6K on Thr421/Ser424 was decreased in ZR-82 cells, while it tended to be decreased in ZR-78.1C and increased in ZR-87 cells. These data suggest that mTORC1 activity is inherently lower in peroxisome-deficient cell lines. Importantly, in accordance with our data, a recent study suggested that acetyl-CoA derived from peroxisomal β-oxidation promotes Raptor acetylation and mTORC1 activation in hepatocytes, and hepatic Acox1 deficiency resulted in inhibition of mTORC1 (He et al., 2020).

The phosphatidylinositol 3-kinase (PI3K)/AKT pathway is involved in SREBP-2 transport to the Golgi and thereby contributes to the control of SREBP-2 activation (Du et al., 2006). To compare AKT phosphorylation in CHO-K1 and peroxisome-deficient cells, immunoblot analyses were performed on lysates from cells treated with U18666A or cultured in FCS and LPDS for 24 h (Figures 8A–C). AKT phosphorylation on Ser473 was similar in FCS-cultured cells and slightly decreased in LPDS- or U18666A-treated ZR-82 and ZR-87 cells compared with CHO-K1 cells. Phosphorylation of AKT was only increased in LPDS-cultured ZR-78.1C cells.

Major signaling pathways that couple transcription factors to extracellular stimuli include the mitogen-activated protein (MAP) kinase cascades through extracellular signal-regulated kinase (ERK1/2). It has been shown that the mature form of SREBP-2 is a substrate of ERK-MAPK *in vitro*, affecting its trans-activity (Kotzka et al., 2000, 2004). In order to examine a possible link between the MAPK pathway and increased SREBP-2 activity and expression of cholesterol biosynthetic genes, we determined whether the phosphorylation of p44/42 ERK was affected in peroxisome-deficient CHO cells. We measured the amounts of total and phosphorylated ERK proteins in cells treated with U18666A or cultured in the presence of FCS or LPDS for 24 h (Figures 8A–C). Total ERK levels were similar in CHO-K1 and peroxisome-deficient cells; however, phosphorylated ERK levels were equally increased in ZR-82 cells under all three culture conditions.

We conclude that the activities of the PI3K/AKT and mTORC1 pathways are not, in general, increased in peroxisome-deficient cells, and also are not the cause of the observed increased expression of cholesterol biosynthetic genes. That said, however, phosphorylated ERK could contribute to higher activity of SREBP-2 in ZR-82 cells.

### Increased Transfer of GFP-SCAP From the ER to the Golgi in Peroxisome-Deficient CHO Cells

**FIGURE 9:**
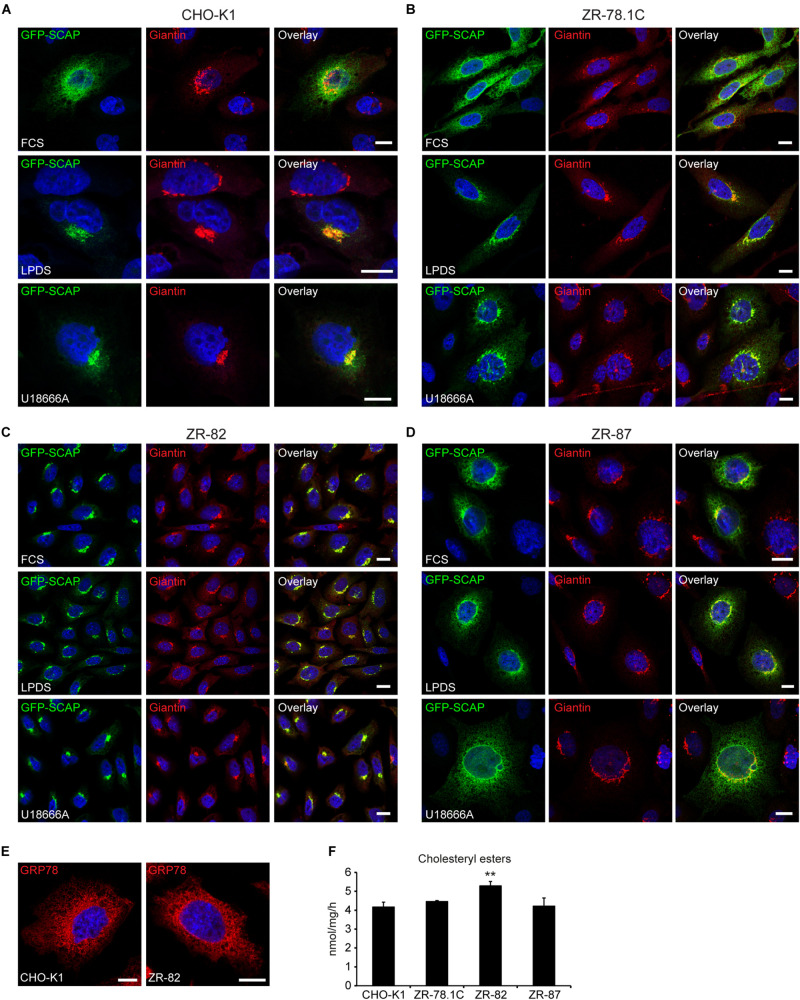
Peroxisome deficiency promotes ER-to-Golgi trafficking of GFP-SCAP. Confocal microscopy images show GFP-SCAP trafficking related to the Golgi marker giantin in CHO-K1 **(A)**, ZR-78.1C **(B)**, ZR-82 **(C)**, and ZR-87 **(D)** cells. Cells were either cultured in 10% FCS or 5% LPDS for 24 h or treated with 10 μM U18666A for 12 h. GFP-SCAP was imaged using a chicken polyclonal anti-GFP antibody followed by Alexa488 secondary antibody; Golgi was visualized by rabbit anti-giantin followed by Alexa594 secondary antibody. The nuclei were stained with DAPI (blue). Representative fields are shown for each condition. The scale bars represent 10 μm. **(E)** The endoplasmic reticulum was detected with an antibody against GRP78. The scale bars represent 10 μm. **(F)** Cholesterol esterification in CHO-K1 and peroxisome-deficient cells. Cells cultured in 10% FCS were incubated for 4 h with [^14^C]palmitate (∼8000 dpm/nmol). Cholesterol ester levels were analyzed by TLC. Data are mean ± SD (*n* = 3). Statistical analysis was performed using one-way ANOVA followed by Dunnett’s multiple comparisons test. **p < 0.01 vs. FCS-cultured CHO-K1.

SCAP, the sterol sensor, plays an essential role in SREBP-2 activation by mediating its ER-to-Golgi transport. To test if peroxisome deficiency enhances ER-to-Golgi trafficking of SCAP/SREBP, we analyzed SCAP trafficking in CHO-K1 and peroxisome-deficient CHO cells using a GFP-SCAP fusion protein, which allows determination of the subcellular distribution of GFP-SCAP by confocal microscopy. Cells stably expressing GFP-SCAP were cultured in medium either containing 10% FCS or 5% LPDS or, alternatively, 10% FCS and 10 μM U18666A (Figures 9A–D). GFP-SCAP showed a reticular ER pattern in CHO-K1 cells grown in cholesterol-containing medium. Incubating cells in medium with 5% LPDS for 24 h or treatment with U18666A for 12 h led to increased juxtanuclear staining and co-immunolocalization with giantin (also known as GOLGB1), a Golgi marker (Linstedt and Hauri, 1993), in addition to the reticular ER pattern (Figure 9A). Peroxisome-deficient ZR-78.1C and ZR-87 cells showed already an increased juxtanuclear staining when cultured in medium containing 10% FCS; incubating cells in medium containing 5% LPDS or treatment with U18666A led to a strong colocalization of GFP-SCAP with giantin (Figures 9B,D). In ZR-82 cells GFP-SCAP localized independently from the culture conditions only to the Golgi (Figure 9C). In general, the data show that deficiency of functional peroxisomes promotes GFP-SCAP trafficking to the Golgi.

To exclude the possibility that the altered GFP-SCAP localization in ZR-82 cells cultured in medium containing 10% FCS was caused by general changes in ER morphology, we stained the ER with an antibody against the glucose-regulated protein 78 (GRP78). However, the morphology of the ER was comparable in CHO-K1 and ZR-82 cells (Figure 9E).

Radhakrishnan et al. (2008) determined the concentration of cholesterol in the ER that is sufficient to retain the SCAP-SREBP-2 complex in the ER and suppress SREBP-2 processing. They showed that SCAP transported SREBP-2 from the ER to the Golgi when ER cholesterol was ≤5 mol% of ER lipids, and ER-to-Golgi transport of SCAP was inhibited when the cholesterol content of the ER rose above the sharp 5% threshold. An open question is whether the cholesterol level in the ER is reduced in peroxisome-deficient cells, which could explain the increased ER-to-Golgi trafficking of SCAP and SREBP-2 activation in peroxisome-deficient cells even when total cholesterol levels are comparable to control cells. Therefore, we determined the rate of cholesterol esterification as a surrogate marker for ER cholesterol levels (Figure 9F). The enzyme acyl-coenzyme A:cholesterol acyltransferase (SOAT) is located in the ER and synthesizes cholesteryl esters, using cholesterol and fatty acyl-CoA as its substrates (Chang et al., 2006). Two *Soat* genes have been identified in mammals (*Soat1* and *Soat2*), and SOAT1 is the major isoenzyme in CHO cells. Assuming that SOAT is located in the ER and that its activity is controlled by cholesterol availability, SOAT activity in intact cells has been used to monitor cholesterol arriving at the ER in cholesterol trafficking studies (Chang et al., 2006). We found that the esterification of cholesterol was similar in CHO-K1 cells and peroxisome-deficient cell lines (Figure 9F), suggesting that ER cholesterol levels are normal in peroxisome-deficient cells.

**FIGURE 10:**
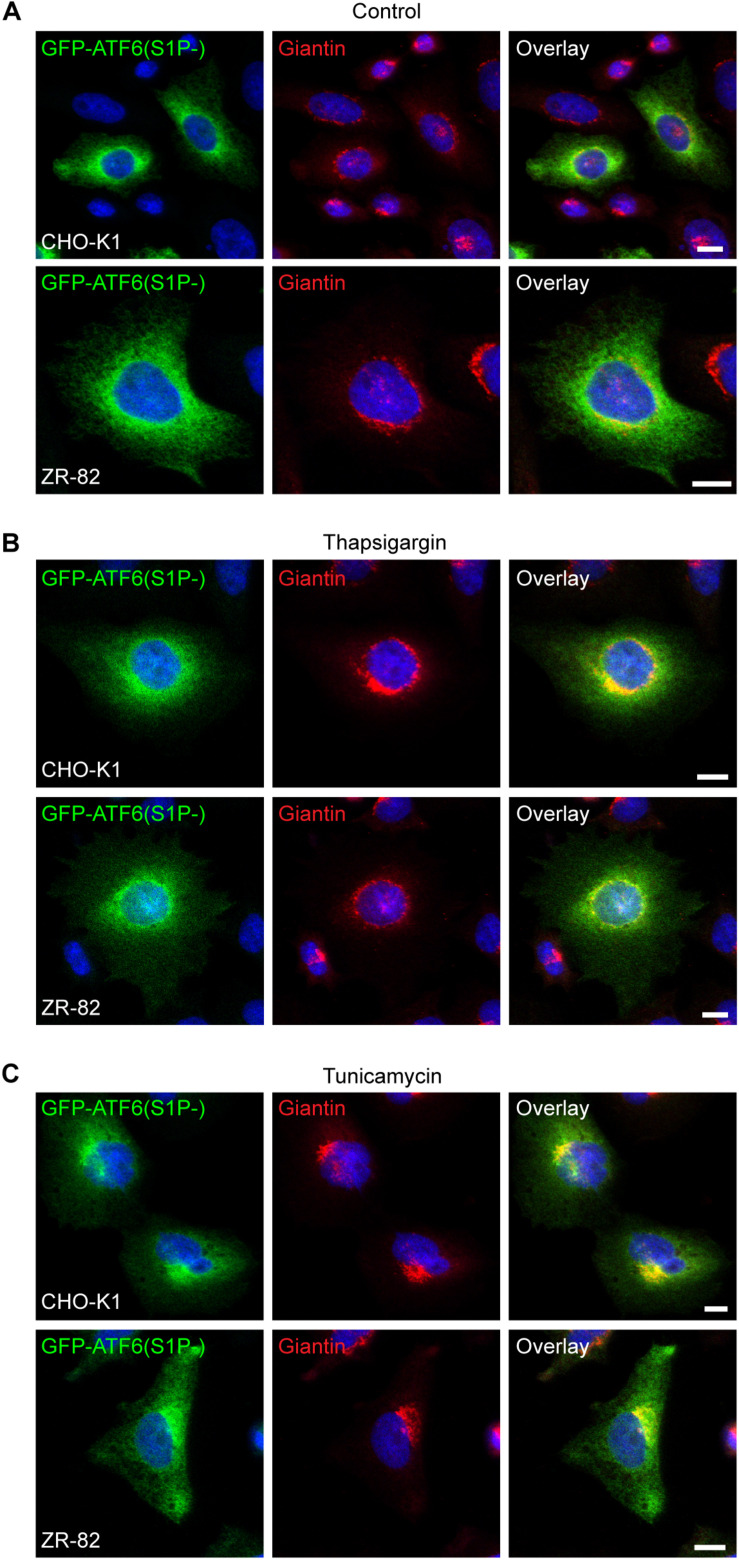
Peroxisome deficiency does not enhance ER-to-Golgi trafficking of GFP-ATF6(S1P-). Confocal microscopy images show GFP-ATF6(S1P-) trafficking related to the Golgi marker giantin in CHO-K1 and ZR-82 cells. Cells were either cultured in 10% FCS (Control) **(A)** or treated with 100 nM thapsigargin **(B)** or 0.5 μg/ml tunicamycin **(C)** for 12 h. GFP-ATF6 was visualized using a chicken polyclonal anti-GFP antibody; the Golgi was visualized by rabbit anti-giantin; DAPI was used for nuclear staining. Representative fields are shown for each condition. The scale bars represent 10 μm.

To exclude the possibility that the altered GFP-SCAP localization in ZR-82 cells cultured in medium containing 10% FCS was caused by general changes in ER morphology, we examined the localization of activating transcription factor 6 (ATF6) in CHO-K1 and ZR-82 cells. ATF6 resides as a transcriptionally inactive membrane-bound precursor protein in the ER by its interaction with GRP78. In response to ER stress and dissociation of GRP78, ATF6 is trafficked from the ER to the Golgi where it is sequentially cleaved by the proteases S1P and S2P to produce an active transcription factor, a process similar to that involving SREBP-2 (Ye et al., 2000; Chen et al., 2002). However, SCAP is not required for the ER-to-Golgi translocation of ATF6, but a similar molecular escort may bind ATF6 (Ye et al., 2000; Chen et al., 2002). We transfected CHO-K1 and ZR-82 cells with a GFP-ATF6 construct where the S1P site was mutated [GFP-ATF6(S1P-)] (Chen et al., 2002). The S1P cleavage blockade due to the S1P site mutation causes a longer retention of GFP-ATF6 in the Golgi after ER stress-induced ER-to-Golgi transfer (Chen et al., 2002). The cells were analyzed by fluorescence microscopy and the staining of GFP-ATF6(S1P-) in the perinuclear and cytoplasmic regions of FCS-cultured CHO-K1 and ZR-82 cells was consistent with ER localization (Figure 10A). Treatment with thapsigargin (Figure 10B) and tunicamycin (Figure 10C) for 12 h caused the movement of GFP-ATF6(S1P-) to perinuclear locations consistent with a Golgi marker staining pattern. GFP-ATF6(S1P-) colocalized with the Golgi marker giantin in both cell lines in response to ER stress induction (Figures 10B,C), but GFP-ATF6(S1P-) was still detectable in the ER in both cell lines. These data show that the altered SCAP localization in peroxisome-deficient cells is specific for SCAP, while other proteins normally subject to ER-to-Golgi transport were not affected, indicating that defective ER-to-Golgi transport is not a generalized phenomenon.

**FIGURE 11:**
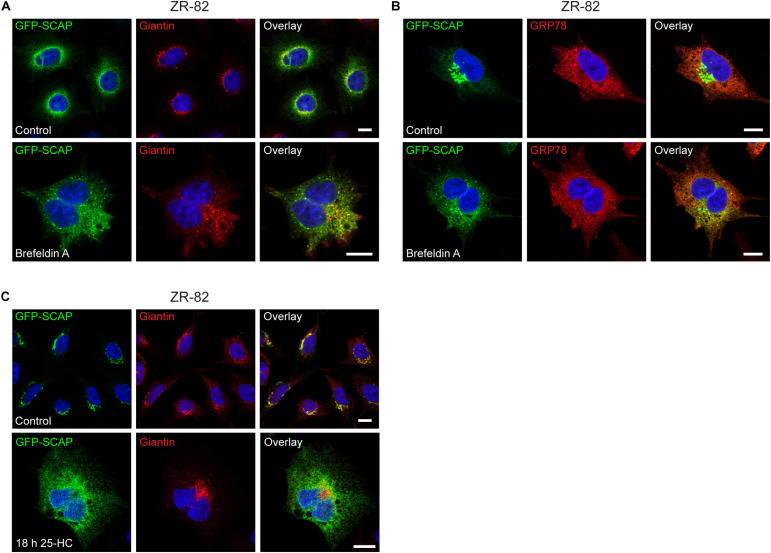
Brefeldin A and 25-HC sensitivity of GFP-SCAP localization in ZR-82 cells. **(A,B)** ZR-82 cells transfected with *GFP-SCAP* were treated with brefeldin A (5 μg/ml) for 30 min. **(A)** Cells were immunostained for GFP-SCAP using a chicken polyclonal anti-GFP antibody and giantin. **(B)** Cells were immunostained for GFP-SCAP and GRP78. **(C)** ZR-82 cells transfected with *GFP-SCAP* were treated with 10 μg/ml 25-HC for 18 h and immunostained for GFP-SCAP and giantin. DAPI was used for nuclear staining. Representative fields are shown for each condition. The scale bars represent 10 μm.

Normally, SCAP is retrieved from the Golgi and moves back to the ER by COPI-mediated Golgi-to-ER retrograde trafficking after processing of SREBP under sterol-deficient conditions (Takashima et al., 2015). To provide further evidence that SCAP in peroxisome-deficient cells increasingly moves to the Golgi, we used brefeldin A, which causes the relocation of Golgi proteins into the ER (Lippincott-Schwartz et al., 1989; Klausner et al., 1992). GFP-SCAP-transfected ZR-82 cells cultured in medium containing 10% FCS were treated with brefeldin A for 30, 60, and 90 min. GFP-SCAP showed a Golgi-like staining pattern and co-localization with giantin in ZR-82 cells cultured in medium containing 10% FCS (Figure 11A). Even by 30 min, treatment with brefeldin A caused the pattern to disperse and return to a more ER-like pattern (Figure 11A). However, as expected, the staining pattern of the ER marker GRP78 was not affected by brefeldin A treatment (Figure 11B).

Next, we examined if treatment with 25-HC affects the GFP-SCAP localization in ZR-82 cells. We have shown that 25-HC suppressed both cholesterogenic promoter activity in LPDS-treated (Figure 3B) and activation of SREBP-2 target genes in U18666A-treated (Figure 5A) CHO-K1 and peroxisome-deficient cells. GFP-SCAP-transfected ZR-82 cells cultured in medium containing 10% FCS were treated with 10 μg/ml 25-HC for 90 min, 12, 15, and 18 h. GFP-SCAP showed a Golgi-like staining pattern and co-localization with giantin in vehicle-treated ZR-82 cells (Figure 11C) and cells treated with 25-HC for 90 min (data not shown). Treatment with 25-HC for 12 and 15 h caused the pattern to disperse and return to a more ER-like pattern (data not shown), and GFP-SCAP showed a reticular ER pattern after an 18 h treatment with 25-HC (Figure 11C).

### Restoring Functional Peroxisomes Normalized the Transcriptional Regulation of the Cholesterol Biosynthetic Pathway, the Rate of Cholesterol Synthesis, and SCAP Trafficking

**FIGURE 12:**
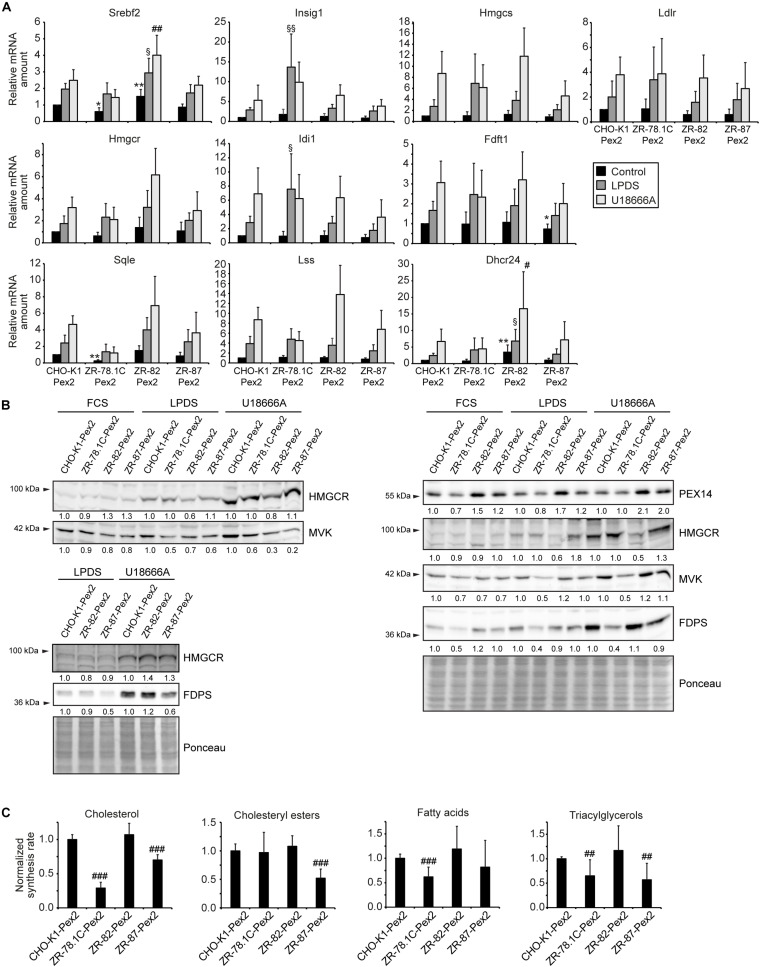
Restoring functional peroxisomes normalized the transcriptional regulation of the cholesterol biosynthetic pathway, the rate of cholesterol synthesis, and SCAP trafficking. **(A)** Expression of genes involved in cholesterol biosynthesis and its regulation in *Pex2*-transfected CHO-K1 and *Pex2*-complemented peroxisome-deficient CHO cells (ZR-78.1C-Pex2, ZR-82-Pex2, ZR-87-Pex2) cultured in medium containing 10% FCS, 5% LPDS and 10 μM U18666A for 24 h. Each value represents the amount of mRNA relative to that in FCS-cultured CHO-K1-Pex2, which was arbitrarily defined as 1. Data are mean ± SD (*n* = 6). **(B)** Immunoblots of total cell lysates with antibodies against cholesterol biosynthetic enzymes after culturing cells in medium containing 10% FCS, 5% LPDS and 10 μM U18666A for 24 h. **(C)** Rate of biosynthesis of cholesterol, cholesteryl esters, fatty acids, and triacylglycerols in vehicle- and U18666A-treated CHO-K1-Pex2 and *Pex2*-complemented peroxisome-deficient CHO cells (ZR-78.1C-Pex2, ZR-82-Pex2, ZR-87-Pex2) as measured by the incorporation of [^14^C]acetate. Lipids were separated by thin-layer chromatography after [^14^C]acetate labeling. Data are mean ± SD (*n* = 9). Statistical analysis was performed using one-way ANOVA followed by Dunnett’s multiple comparisons test. *p < 0.05; **p < 0.01; ***p < 0.001 vs. FCS-cultured CHO-K1-Pex2. ^#^*p* < 0.05; ^##^*p* < 0.01; ^###^*p* < 0.001 vs. U18666A-cultured CHO-K1-Pex2. ^§^ *p* < 0.05; ^§§^ *p* < 0.01; ^§§§^ *p* < 0.001 vs. LPDS-cultured CHO-K1-Pex2.

To examine whether restoring functional peroxisomes normalizes the transcriptional regulation of the cholesterol biosynthetic pathway, the expression of SREBP-2 target genes was determined in *Pex2*-transfected cells cultured in medium containing 10% FCS, 5% LPDS, or 10% FCS plus 10 μM U18666A for 24 h (Figure 12A). Incubation in medium containing 5% LPDS or 10 μM U18666A significantly increased the mRNA levels of SREBP-2 target genes in all cell lines compared with FCS-cultured cells. However, the expression levels in CHO-K1-Pex2 cells and *Pex2*-complemented ZR cells with restored functional peroxisomes were similar under all conditions. Next, the protein levels of HMGCR, MVK, and FDPS were determined in CHO-K1-Pex2 and *Pex2*-complemented ZR cell lines cultured in medium containing 10% FCS, 5% LPDS, or 10 μM U18666A for 24 h (Figure 12B). The cultivation of the cells in medium containing 5% LPDS and the treatment with U18666A increased the protein levels of HMGCR, MVK and FDPS in all cell lines as expected. MVK and FDPS protein levels tended to be lower in ZR-78.1C-Pex2 cells, whereas HMGCR protein levels tended to be lower in LPDS-cultured and U18666A-treated ZR-82-Pex2 cells (Figure 12B). Interestingly, the protein levels of PEX14 were increased in *Pex2*-transfected ZR-82 cells. In summary, compared to peroxisome-deficient cells, no significant increase in protein levels of cholesterol biosynthesis enzymes compared to CHO-K1 cells was observed in *Pex2*-transfected ZR cells after restoring peroxisome functionality.

To assess whether restoring functional peroxisomes in ZR cells also normalizes *de novo* cholesterol biosynthesis, we measured *de novo* lipogenesis by quantifying incorporation of [^14^C]acetate into lipids in CHO-K1-Pex2, ZR-78.1C-Pex2, ZR-82-Pex2, and ZR-87-Pex2 cells cultured in cholesterol-containing medium with 10 μM U18666A. *De novo* cholesterol synthesis was similar in CHO-K1-Pex2 and ZR-82-Pex2 cells and significantly increased in ZR-87-Pex2 cells, whereas cholesterol synthesis was still significantly decreased in ZR-78.1C-Pex2 cells (Figure 12C). However, mutant ZR-78.1C, in contrast to ZR-82 and ZR-87 cells, have secondary mutations, are ouabain-resistant, and hypoxanthine phosphoribosyltransferase (HGPRT)-negative in hybridization studies (Ralph Zoeller, personal communication); hence, these alterations might prevent the normalization of cholesterol synthesis in ZR-78.1C-Pex2 cells, even though the dysregulation of the SREBP-2 pathway is corrected.

**FIGURE 13:**
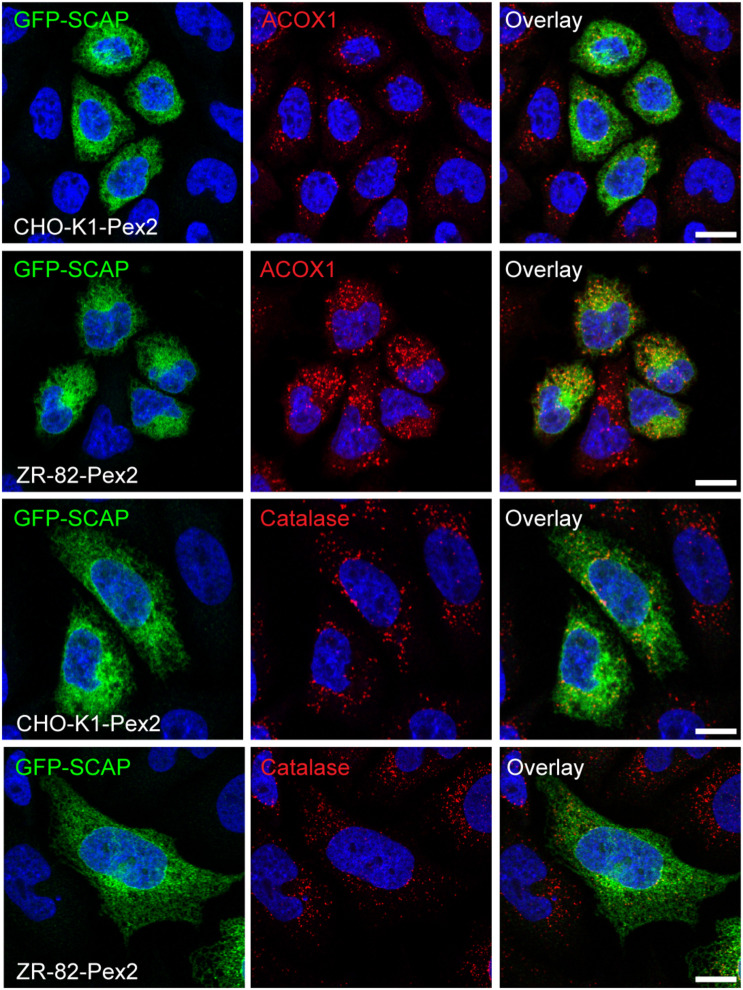
Restoring functional peroxisomes in ZR-82 cells prevents enhanced ER-to-Golgi trafficking of GFP-SCAP. CHO-K1-Pex2 and ZR-82-Pex2 cells were transiently transfected with *GFP-SCAP* and cultured in 10% FCS. GFP-SCAP was imaged using a chicken polyclonal anti-GFP antibody followed by Alexa488 secondary antibody; peroxisomes were visualized by rabbit anti-ACOX1 and rabbit anti-catalase followed by Alexa594 secondary antibody; DAPI was used for nuclear staining. The scale bars represent 10 μm.

Finally, we examined if the enhanced localization of GFP-SCAP to the Golgi in peroxisome-deficient cells could be prevented by restoring functional peroxisomes. Since GFP-SCAP localized independently of culture conditions exclusively to the Golgi complex in ZR-82 cells, ZR-82-Pex2 cells were transiently transfected with GFP-SCAP. GFP-SCAP showed a reticular ER pattern both in CHO-K1-Pex2 and ZR-82-Pex2 cells grown in cholesterol-containing medium (Figure 13). Importantly, none of the transfected ZR-82-Pex2 cells showed localization of GFP-SCAP to the Golgi complex.

Taken together, restoring functional peroxisomes in these cells resulted in a coordinated transcriptional and post-transcriptional regulation of the cholesterol biosynthetic pathway, demonstrating the importance of peroxisome functionality for this pathway.

## Discussion

Herein, we have provided evidence that functional peroxisomes are necessary for efficient cholesterol synthesis and that peroxisome deficiency dysregulates the SREBP-2 pathway. Cells maintain cholesterol homeostasis by multiple feedback controls that act through transcriptional and post-transcriptional mechanisms, especially transcriptionally through SREBPs (Goldstein et al., 2006) and sterol-accelerated ubiquitination and degradation of HMGCR (DeBose-Boyd, 2008). Cholesterol synthesis and uptake are tightly regulated by SREBP-2 (Horton et al., 2003), a membrane-bound transcription factor that undergoes sterol-regulated export from the ER to the Golgi apparatus for proteolytic processing. The transcriptionally inactive SREBP-2 precursor protein is retained in the ER membrane through the interaction with SCAP and INSIG proteins. In sterol-depleted cells, such as would occur under conditions of starvation or where the extracellular supply of cholesterol is diminished substantially, SCAP undergoes a conformational change and it escorts SREBP-2 from the ER to the Golgi in COPII vesicles, where it is processed to its mature form. The ER contains relatively low levels of sterols compared to the whole cell or plasma membrane, making it a highly effective environment for the high-affinity cholesterol sensor SCAP to reside (Brown et al., 2018). Radhakrishnan et al. (2008) showed that SCAP transports SREBP-2 to the Golgi when the ER cholesterol content was less than 5 mol% of the total ER lipids. SCAP transport was inhibited when cholesterol content of ER membranes rose above this minimum threshold. The SREBP-2 pathway is activated in peroxisome-deficient CHO cells even when grown in cholesterol-replete medium. Accordingly, stronger activation of the SREBP-2 pathway is seen when peroxisome-deficient CHO cells are grown in cholesterol-deficient medium or if cholesterol is sequestered in the lysosomes, e.g., upon U18666A treatment. However, peroxisome-deficient cells are still sensitive towards 25-HC, indicating that the sterol-dependent mechanism for activation of the SREBP-2 pathway is still intact.

What is the cause of the increased transfer of SCAP from the ER to the Golgi and the dysregulation of the SREBP-2 pathway in peroxisome-deficient cells? Dysregulation of SREBP activation has been associated with ER stress and activation of the unfolded protein response (Colgan et al., 2011). In contrast to observations in the liver and extrahepatic tissues of peroxisome-deficient *Pex2*^–/–^ mice (Kovacs et al., 2009, 2012; Figures 6F–J), we did not observe an induction of ER stress markers in peroxisome-deficient CHO cells; this might be due to adaptation of the stable cell lines during the course of propagation. Another explanation for why no ER stress and UPR activation was observed in peroxisome-deficient CHO cells, in contrast to, e.g., postnatal *Pex2*^–/–^ mouse tissues or biopsies obtained from patients with peroxisomal disorders (Kovacs et al., 2009, 2012; Launay et al., 2017), might be the lack of accumulation of ER stress-inducing metabolites. Indeed, it has been shown that ER stress and UPR are induced in human primary skin fibroblast cell lines from X-linked adrenoleukodystrophy patients with *ABCD1* mutations and impaired peroxisomal β-oxidation only after incubation with very long-chain fatty acids (van de Beek et al., 2017). All three parallel branches of the UPR− governed by the ER-stress sensors IRE1, PERK, and ATF6, respectively, are activated upon pharmacologically induced ER stress (Lin et al., 2007; Zhang et al., 2015). However, also acute ER stress, which was chemically induced in the *in vitro* system used in the present study, did not activate SREBP-2 in CHO-K1 cells.

Mechanistic target of rapamycin complex 1 has emerged as a regulator of lipid biosynthesis (Laplante and Sabatini, 2012). Porstmann et al. (2005) showed that activation of Akt, an upstream activator of mTOR, induced the expression of SREBP-1 and its target genes, but the effect on SREBP-2 was less pronounced. The mTOR inhibitor rapamycin has been shown to have opposing effects on SREBP-2 processing and the expression of HMGCR in different cell types (Gueguen et al., 2007; Sharpe and Brown, 2008; Ma et al., 2010). It has been shown that SREBP-2 processing and cholesterol biosynthetic gene expression are coordinately regulated by the eukaryotic initiation factor 4E-binding protein 1 (4E-BP1) and p70 S6K, and that the relative contribution of each mTORC1 effector varies between cell types (Wang et al., 2011). It has been reported that an increase in mTORC1 activity is accompanied by a decrease in ER cholesterol and by SREBP-2 activation (Eid et al., 2017). mTORC1, through its regulation of autophagy and endosomal membrane trafficking, activates SREBP-2 by suppressing cholesterol trafficking to lysosomes in mammalian cells (Eid et al., 2017). On the other hand, cholesterol has been shown to drive mTORC1 recruitment and activation at the lysosomal surface through a mechanism that is, at least in part, distinct from growth factor signaling (Castellano et al., 2017). Our data suggest that mTORC1 does not play a role in SREBP-2 activation or in the increased expression of cholesterol biosynthetic genes in peroxisome-deficient CHO cells, since the mTORC1 activity is inherently lower in peroxisome-deficient cell lines (i.e., decreased phosphorylation of S6 and p70 S6K). However, we cannot exclude the possibility that another target of mTORC1 could be involved in the activation of SREBP-2 in CHO cells instead of p70 S6K. Importantly, in accordance with our data, a recent study suggested that acetyl-CoA derived from peroxisomal β-oxidation promotes Raptor acetylation and mTORC1 activation in hepatocytes, and hepatic Acox1 deficiency resulted in inhibition of mTORC1 (He et al., 2020).

Despite the sensitivity of the SREBP-2 pathway to ER cholesterol levels, the rate of cholesterol synthesis is lower in peroxisome-deficient cells compared with CHO-K1 cells, illustrating the fact that functional peroxisomes and probably the compartmentalization of cholesterol biosynthetic enzymes in peroxisomes are necessary to ensure optimal biosynthesis of cholesterol. Consequently, plasma cholesterol levels are reduced both in patients with peroxisome biogenesis disorders and in 10-day-old *Pex2*^–/–^ mice (Kovacs et al., 2002, 2004). Indeed, restoring functional peroxisomes by complementing peroxisome-deficient CHO cells with rat *Pex2* cDNA enabled efficient cholesterol synthesis, at levels comparable to those of control (CHO-K1) cells.

Interestingly, loss of intact peroxisomes leads to enhanced ER-to-Golgi trafficking of GFP-SCAP. SCAP trafficking was normalized after restoration of functional peroxisomes, which goes also hand in hand with a normalization of the expression of SREBP-2 target genes and the rate of cholesterol synthesis. Thus, intact peroxisomes are a prerequisite for efficient cholesterol synthesis and their loss leads to defective cholesterol homeostasis.

Peroxisome-deficient ZR-78.1C, ZR-82, and ZR-87 cells have mutations in *Pex2*, a gene encoding an integral peroxisomal membrane protein, resulting in nonfunctional PEX2 (Zoeller et al., 1989; Thieringer and Raetz, 1993). PEX2 is involved in the translocation of peroxisomal matrix proteins following the docking of PTS receptors to the peroxisome membrane. These mutant cells exhibit typical peroxisomal membrane “ghosts”− abnormal membrane-delimited compartments that contain integral peroxisomal membrane proteins, but lack peroxisomal matrix proteins (Santos et al., 1988, 2000). In such cases (e.g., Zellwegers Syndrome, and other peroxisomal deficiency diseases), peroxisomal matrix proteins such as catalase and ACOX1 are not appropriately compartmentalized in peroxisomes; instead, they are diffusely distributed throughout the cytoplasm, consistent with the inability of these mutant cells to import peroxisomal matrix proteins (Figure 2). The number of peroxisomal ghosts in the peroxisome-deficient cells is lower compared with the number of functional peroxisomes in CHO-K1 cells, however, the number of ghosts also varies between the peroxisome-deficient cells Figures 2B,C). Recently, tethers between the ER and peroxisomes have been identified in mammalian cells, involving the peroxisomal tail-anchored proteins ACBD4 and ACBD5 and the ER-resident vesicle-associated membrane protein-associated proteins (VAPs) (Costello et al., 2017a,b; Hua et al., 2017). In immunostaining experiments in peroxisome-deficient cells, ACBD5 and PEX14 were present in cellular vesicles, suggesting that membrane contact sites between peroxisomal membrane ghosts and the ER might exist in peroxisome-deficient CHO cells (Figures 2B, C). Hua et al. (2017) showed that tethering of peroxisomes to the ER via ACBD5-VAP-mediated contact sites is necessary for the maintenance of cellular cholesterol and plasmalogen levels: depletion of these tethers resulted in a decrease of total cholesterol levels. While total cholesterol levels in peroxisome-deficient CHO cells were normal, it is tempting to speculate that a transfer of farnesyl diphosphate to the ER in order to be used as substrate of FDFT1 might occur via ACBD5-VAP-mediated contact sites.

The cholesterol transfer from endocytic organelles to the ER likely involves several parallel pathways (Pfisterer et al., 2016). Recently, it has been suggested that LDL-derived cholesterol traffics from lysosomes via peroxisomes to the ER and that membrane contact sites facilitate this cholesterol transfer (Chu et al., 2015; Hu et al., 2018; Xiao et al., 2019). Tethering between the lysosomes and peroxisomes is facilitated by peroxisomal phosphatidylinositol-4,5-biphosphate (PI(4,5)P_2_) and synaptotagmin 7 on lysosomes, and disruption of the lysosome-peroxisome membrane contacts caused cholesterol accumulation in lysosomes (Chu et al., 2015). Since ZR-78.1C, ZR-82, and ZR-87 cells lack functional peroxisomes, but contain abundant peroxisomal membrane ghosts, lysosome-peroxisome membrane contact sites might be able to form in such cells. This raises a fundamental biological question: if cholesterol traffics from the lysosome to peroxisomes for further distribution in the cell, are peroxisomal matrix proteins necessary (i.e., must the cholesterol go into the matrix) or does the transfer occur only via the peroxisomal membrane? Here, we demonstrated that total cholesterol levels were similar in CHO-K1 and peroxisome-deficient CHO cells. Interestingly, we and others have reported that cholesterol levels are either unaffected or decreased in peroxisome biogenesis-defective human fibroblasts, mouse tissues or serum (Kovacs et al., 2002; Faust and Kovacs, 2014). These findings are at odds with those of Chu et al. (2015); the reasons for this disparity are not yet clear. Cholesterol is distributed among multiple cellular pools, and any newly appearing cholesterol (arising either by *de novo* synthesis or by extracellular uptake) is rapidly incorporated into many types of cellular membranes by both vesicular and non-vesicular means (Lange and Steck, 2016). It might be that peroxisomal dysfunction results in subcellular cholesterol redistribution without a net increase in cholesterol content. Furthermore, we cannot exclude the possibility that peroxisome deficiency alters the equilibrium among different cellular cholesterol pools. In particular, cholesterol represents only about 5% of the total lipid mass of ER membranes, making them exquisitely sensitive to changes in the levels of cholesterol in cells (Radhakrishnan et al., 2008). However, peroxisome-deficient CHO cells are sensitive to inhibition of SREBP-2 activation by 25-HC, which enters cells and reaches the ER without traversing the lysosomes (and by a mechanism that does not involve membrane vesicles). This suggest that the regulation of SREBP-2 processing via hydroxysterols is still responsive in peroxisome-deficient cells. However, peroxisome deficiency leads to relocalization of SCAP to the Golgi, thereby altering the SCAP-Insig-SREBP-2 cholesterol sensing complex. In conclusion, functional peroxisomes are essential for efficient cholesterol sensing and synthesis.

The current experiments were performed not only in medium containing LPDS, but also in the presence of 10% serum (FCS), which provides an abundant source of cholesterol and fatty acids and usually suppresses the activation of all three SREBPs (Hannah et al., 2001). Despite the presence of lipid-replete medium, peroxisome deficiency led to activation of SREBP-specific reporter genes and increased expression of SREBP target genes. However, as mentioned above, it is not known whether cholesterol obligatorily enters the peroxisomal matrix in order to be distributed to other intracellular compartments or if the redistribution only occurs via the peroxisomal membrane. Blocking the exit of cholesterol from the lysosomes by treatment with U18666A resulted in activation of the SREBP-2 pathway, both in CHO-K1 and peroxisome-deficient cells, however, the increase of SREBP-2 target genes was more pronounced in peroxisome-deficient CHO cells, whereas cholesterol synthesis was lower in these cell lines.

Taken together, our findings demonstrate that disruption of peroxisomal function directly leads to a disarray in cholesterol metabolism and regulation, despite normal cholesterol content and sensitivity to external stimuli via 25-HC. Thus, intact (functional) peroxisomes are of utmost importance for a balance in the cholesterol homeostatic pathways.

## Materials and Methods

### Materials

All chemicals were reagent grade and were purchased from Sigma/Aldrich (St. Louis, MO) unless otherwise indicated. Fetal bovine lipoprotein-deficient serum (LPDS) was obtained from Intracel (Frederick, MD, United States) or Sigma (S5394). U18666A (3-β-[2-(diethylamino)ethoxy]androst-5-en-17-one) (BML-S200; Enzo Life Sciences, Switzerland) was dissolved in ethanol and stored as a 10 mM stock solution at −20°C. 25-hydroxycholesterol (25-HC) (H1015; Sigma/Aldrich) was dissolved in ethanol as a 10 mg/ml stock solution and stored at −20°C. Tunicamycin (T7765; Sigma/Aldrich) was dissolved in DMSO as a 4 mg/ml stock solution and stored at −20°C. Thapsigargin (BML-PE180; Enzo Life Sciences) was dissolved in DMSO as a 1 mM stock solution and stored at −20°C. Brefeldin A (B6542; Sigma/Aldrich) was dissolved in DMSO as 10 mg/ml stock solution and stored at −20°C.

### Cell Culture

The CHO cell line, CHO-K1, and the peroxisome-deficient mutant CHO lines ZR-78.1C, ZR-82, and ZR-87 were obtained from Dr. Raphael A. Zoeller (Boston University School of Medicine, Boston, MA, United States) (Zoeller and Raetz, 1986). The mutant CHO cell lines were isolated from the CHO-K1 cell line used as control in this study. Cells were maintained in monolayer culture at 37 °C in 5% CO_2_ in Ham’s F-12 medium (Cat. no. 21765-029; Life Technologies, United Kingdom) containing 100 units/ml penicillin and 100 μg/ml streptomycin sulfate supplemented with 10% (v/v) fetal calf serum (FCS).

### Animals

Homozygous *Pex2*^–/–^ mice were obtained by breeding *Pex2* heterozygotes (*Pex2^+/–^*) on a hybrid Swiss Webster-129 (SW/129) background (Kovacs et al., 2004, 2009). Mice had access to food and water *ad libitum* and were exposed to a 12:12-hour light-dark cycle. For the purposes of this study, control mice consisted of *Pex2^+/+^* and *Pex2^+/–^* genotypes (hereafter referred to as *Pex2^+/^*), as their biochemical characteristics were comparable to one another (Kovacs et al., 2004, 2009). All protocols for animal use and experiments were reviewed and approved by the Institutional Animal Care and Use Committee of San Diego State University.

### Transient Transfections of Promoter-Luciferase Reporters

The *Firefly* luciferase gene reporter plasmids for Hmgcr (pRED*luc*, hereafter called HMGCR-Luc) (Vallett et al., 1996) and Srebf2 (-4316/SREBP-2.Luc, hereafter called Srebf2-Luc) (Shin and Osborne, 2003), Fdps (pGL2-FPPS, hereafter called Fdps-Luc) (Ericsson et al., 1996), and Fdft1 (pHSS1kb-Luc, hereafter called Fdft1-Luc) (Guan et al., 1995) promoters were kind gifts from Tim Osborne (University of California, Irvine, CA, United States), Peter Edwards (University of California, Los Angeles, CA, United States), and Ishaiahu Shechter (Uniformed Services University of the Health Sciences, Bethesda, MD, United States), respectively.

Transient transfections were performed in 12-well plates using the Lipofectamine 2000 reagent (Invitrogen, Carlsbad, CA, United States) according to the manufacturer’s instructions. Cells were seeded at a density of 5 × 10^4^ cells/well. Cells were transfected with 500 ng of the luciferase reporter plasmids. Transfection efficiencies were normalized by co-transfecting in the ratio of 1:10 with a control plasmid encoding *Renilla* luciferase driven by the HSV-thymidine kinase promoter (pRL-TK, Cat. no. E2241, Promega, Madison, WI, United States). After transfection cells were cultured in medium with either 10% FCS or 5% LPDS for 24 h. After incubation cells were washed twice with PBS and harvested in 1× Passive Lysis Buffer (Promega). *Firefly* and *Renilla* luciferase activities in the cell lysates were measured with the Dual-Luciferase^®^ reporter assay system (Promega) according to the manufacturer’s protocol. Photon production was detected as relative light units by using an Analytical Luminescence Laboratory Monolight^TM^ 2010 Luminometer. The values presented are the means of at least three separate transfections done in quadruplicate. The amount of *Firefly* luciferase activity in relative light units was normalized to the amount of *Renilla* luciferase activity from the same test tube.

### Western Blot Analysis

Cells were washed 3x in phosphate buffered saline (PBS) and lysed in RIPA buffer (20 mM Tris, pH 7.5; 150 mM NaCl; 1 mM EDTA; 1 mM EGTA; 1% NP-40; 1% sodium deoxycholate) containing protease and phosphatase inhibitors (cOmplete and PhosSTOP, respectively, Roche Diagnostics, Mannheim, Germany). Lysates were incubated on ice for 30 min and centrifuged at 20,000 × *g* for 20 min at 4 °C. Protein concentration was determined by the BCA method (Pierce, Rockford, IL, United States). Equal amounts of protein were subjected to SDS-polyacrylamide gel electrophoresis (SDS-PAGE) and electrophoretically transferred to Amersham Protran Supported 0.2 μM nitrocellulose (#10600015; GE Healthcare, Glattbrugg, Switzerland). After blocking for 1 h in TBST (Tris-buffered saline with 0.05% Tween 20) containing 1% bovine serum albumin (BSA), membranes were probed with the indicated primary antibodies (see section “Results” above) overnight at 4°C. The membranes were incubated with horseradish peroxidase-conjugated secondary antibodies (Goat anti-rabbit (#401393) and goat anti-mouse (#401253) from Sigma-Aldrich; goat anti-rat (#629520) and rabbit anti-goat (#611620) from Invitrogen) and were visualized using enhanced chemiluminescence. Membranes were exposed either to Super RX autoradiographic films (Fuji, Düsseldorf, Germany) or the Fusion Solo S imaging system. Antibodies are listed in Supplementary Table S2. Blots were semi-quantitatively analyzed by densitometry using ImageJ 1.52 v (National Institutes of Health).

Immunoblot analysis of HMG-CoA reductase was performed as described by Kovacs et al. (2004). Briefly, trichloroacetic acid-precipitated proteins were first resuspended in 25 μl Tris–HCl (125 mM, pH 6.8), 1% SDS, 0.1 M NaOH, followed by 125 μl of sample buffer containing 7 M urea, 8% SDS, and 1.1 M 2β-mercaptoethanol, and separated on 7.5% acrylamide gels.

### Enzyme Assays

3-hydroxy-3-methylglutaryl (HMG)-CoA reductase (HMGCR; EC 1.1.1.34), farnesyldiphosphate synthase (FDPS; EC 2.5.1.1), and isopentenyldiphosphate isomerase (IDI1; EC 5.3.3.2) activities were assayed as described previously (Kovacs et al., 2004).

### Quantitative Real-Time RT-PCR

Total RNA was prepared from cells and frozen tissues with RNeasy Mini Kit (QIAGEN, Hilden, Germany) and treated with DNase I. Quantitative RT-PCR (qRT-PCR) assays were performed as described previously (Kovacs et al., 2012). First-strand cDNA was synthesized with random hexamer primers using Ready-To-Go You-Prime First-Strand Beads (GE Healthcare, Glattbrugg, Switzerland) and the High-Capacity RNA-to-cDNA Kit (No. 4368813; Applied Biosystems). Real-time RT-PCR was performed on a Roche LightCycler LC480 instrument in duplicates using 10 ng cDNA, 7.5 pmol forward and reverse primers, and the 2x KAPA SYBR FAST qPCR Mastermix (No. KK4601; KAPA Biosystems). Thermal cycling was carried out with a 5 min denaturation step at 95°C, followed by 45 three-step cycles: 10 s at 95°C, 10 s at 60°C, and 10 s at 72°C. Melt curve analysis was carried out to confirm the specific amplification of a target gene and absence of primer dimers. Primer sequences are listed in Supplementary Table S3. Relative mRNA amount was calculated using the comparative threshold cycle (C_*T*_) method (Livak and Schmittgen, 2001). *Gapdh* and *actin* were used as the invariant control for CHO cells, and *18S rRNA* and *cyclophilin* (*Ppia*) were used as the invariant control for mouse tissues.

### Cells Stably Expressing GFP-SCAP, GFP-ATF6(S1P-) and Pex2

pGFP-SCAP plasmid, encoding GFP fused to wild-type hamster SCAP under the control of the CMV promoter, was a gift from Dr. Peter Espenshade (Johns Hopkins University School of Medicine, Baltimore, MD, United States) (Nohturfft et al., 2000). pEGFP-ATF6-(S1P-) was a gift from Ron Prywes (Addgene plasmid # 32956) (Chen et al., 2002). CHO-K1, ZR-78.1C, ZR-82, and ZR-87 cells stably expressing GFP-SCAP were generated by transfection with pGFP-SCAP using polyethylenimine (PEI), followed by selection with G418. After selection with G418 GFP-SCAP-positive cells were sorted on a BD FACSAria^TM^ IIIu BL1 sorter (BD Biosciences). The plasmid (PYN3218) containing full-length rat *Pex2* cDNA was a gift from Masaki Ito (Saga Medical School, Saga, Japan).

### Determination of Rate of Biosynthesis of Lipids

Cells were incubated with 5 μCi [1-^14^C]acetate (58 mCi/mmol; PerkinElmer, Boston, MA, United States) for 4 h. After incubation, the cells were rinsed three times with PBS, and lipids were extracted with hexane:isopropanol (3:2). Manipulative losses of lipids were accounted for by addition of a known amount of [^3^H]cholesterol as internal standard. After lipid extraction cells were lysed in 0.1 M NaOH for protein determination using the BCA method. Acetate incorporation into specific lipids was analyzed after separation of lipids by thin-layer chromatography (TLC). Therefore, organic phases were evaporated to dryness under a nitrogen stream. Lipids were resuspended in chloroform and spotted together with appropriate lipid standards on silica gel 60 plates (Merck, Darmstadt, Germany). For separation of neutral lipids, plates were developed in heptanes:diethylether:acetic acid (90:30:1) as solvent. Lipid samples and standards were visualized by iodine vapor. The lipid fractions were scraped from the plate and their radioactive content was determined by liquid scintillation counting. Values were normalized for sample protein content.

After incubation with 5 μCi [1-^14^C]acetate incorporation into cholesterol was also analyzed by reverse-phase radio-HPLC as previously described (Kovacs et al., 2004). Briefly, following saponification and petroleum ether extraction, the non-saponifiable lipids were analyzed by reverse-phase radio-HPLC, and the specific activities of the radiolabeled products (e.g., sterols, squalene) were determined.

### Cholesterol Esterification

[1-^14^C]palmitic acid (56.1 mCi/mmol, NEC075H050UC) was purchased from PerkinElmer. A 2.5 mM stock solution of [1-^14^C]palmitic acid was prepared by complexing labeled and unlabeled palmitate to fatty acid-free albumin as described previously (Knobloch et al., 2017). The reaction mixture was prepared by diluting the palmitate/BSA stock with cell culture medium and contained 0.4 μCi [1-^14^C]palmitic acid and 100 μM sodium palmitate in 1 ml medium. Cells were seeded in 6-well plates in medium containing 10% FCS (v/v). The reaction was initiated by replacing the culture medium with 1 ml of the reaction mixture. The cells were incubated for 4 h at 37°C. After incubation, lipids were extracted with hexane:isopropanol (3:2) and cholesterol ester levels were analyzed by TLC as described above for the determination of the rate of lipid biosynthesis.

### Immunofluorescence Staining

Immunofluorescence staining was performed as described previously (Kovacs et al., 2007). As secondary antibodies, Alexa Fluor 488 donkey anti-chicken IgY (#703-545-155; Jackson ImmunoResearch), Alexa Fluor 488 goat anti-rabbit IgG (A11070; Invitrogen), Alexa Fluor 594 goat anti-rabbit IgG (A11072; Invitrogen), and Alexa Fluor 594 donkey anti-goat IgG (A11058; Invitrogen) were used in a dilution of 1:400. Images were taken with a Leica SP8-AOBS confocal laser scanning microscope (Leica Microsystems GmbH, Wetzlar, Germany) using a 63× oil immersion objective and the Leica LAS Lite Software. Acquirement settings were optimized for each experiment, and identical settings were used for all images throughout one experiment. Fluorescent dyes were imaged sequentially in frame interlace mode to eliminate crosstalk between the channels. Image processing was performed off-line using Fiji software (Schindelin et al., 2012).

### Statistical Analyses

Data are expressed as mean ± SD. When two groups where compared, statistical significance was evaluated by an unpaired, two-tailed Student’s *t*-test or an unpaired, two-tailed Student’s *t*-test with Welch’s correction when variances where significantly different. For multiple group analysis one-way ANOVA followed by Dunnett’s multiple comparisons test was performed. Data were assumed to be normally distributed. Statistical analyses were performed using GraphPad Prism version 8.2.0.

## Data Availability Statement

The raw data supporting the conclusion of this article will be made available by the authors, without undue reservation.

## Ethics Statement

The animal study was reviewed and approved by Institutional Animal Care and Use Committee of San Diego State University.

## Author Contributions

HS and WK: conception and design. KC, JS, PF, SF, HS, and WK: acquisition, analysis, and interpretation of data. PF, SF, and WK: resources. WK: writing – original draft and study supervision. PF, SF, HS, and WK: writing – review and editing. All authors approved the submitted manuscript.

## Conflict of Interest

The authors declare that the research was conducted in the absence of any commercial or financial relationships that could be construed as a potential conflict of interest.

## Abbreviations

25-HC: 25-hydroxycholesterol
Abca1: ATP-binding cassette transporter A1
ATF4: activating transcription factor 4
ATF6: activating transcription factor 6
BAAT: bile acid-coenzyme A:amino acid N-acyltransferase
bHLH: basic helix-loop-helix
Calr: calreticulin
Canx: calnexin
CHO: Chinese hamster ovary
Chop: C/EBP-homologous protein
COPII: coat protein complex II
DHCR24: 24-dehydrocholesterol reductase
Dnajb9: DnaJ heat shock protein family (Hsp40) member B9
ER: endoplasmic reticulum
FASN: fatty acid synthase
FDFT1: squalene synthase
FDPS: farnesyl diphosphate synthase
Gadd34: growth arrest and DNA damage-inducible 34
GRP78: glucose-regulated protein 78
GRP94: glucose-regulated protein 94
HMGCR: 3-hydroxy-3-methylglutaryl-CoA reductase
HMGCS: 3-hydroxy-3-methylglutaryl-CoA synthase
IDI1: isopentenyl diphosphate isomerase 1
INSIG: insulin-induced gene
LDLR: low density lipoprotein receptor
LPDS: lipoprotein-deficient serum
LSS: lanosterol synthase
MVD: mevalonate pyrophosphate decarboxylase
MVK: mevalonate kinase
NPC1: Niemann-Pick C1
PERK: PKR-like endoplasmic reticulum kinase
Pex: peroxin
PMVK: phosphomevalonate kinase
S1P: Site-1 protease
S2P: Site-2 protease
SC4MOL: methylsterol monoxygenase 1
SCAP: SREBP cleavage-activating protein
SQLE: squalene epoxidase
SRE: sterol regulatory element
SREBF2: sterol regulatory element-binding protein 2
SREBP: sterol regulatory element-binding protein
SSD: sterol-sensing domain
WD: tryptophan-aspartate repeat
Xbp1: X-box -binding protein 1.
