# Supplementary material for: Functional Peroxisomes Are Essential for Efficient Cholesterol Sensing and Synthesis
Source: Front Cell Dev Biol. 2020 Nov 6;8:560266. doi: 10.3389/fcell.2020.560266 (PMC7677142; doi:10.3389/fcell.2020.560266)
Supplement: Supplementary file 1 [file Data_Sheet_1.pdf]

SUPPLEMENTAL INFORMATION

Functional peroxisomes are essential for efficient cholesterol sensing and synthesis

Khanichi N. Charles, Janis E. Shackelford, Phyllis L. Faust, Steven J. Fliesler, Herbert Stangl, Werner J. Kovacs

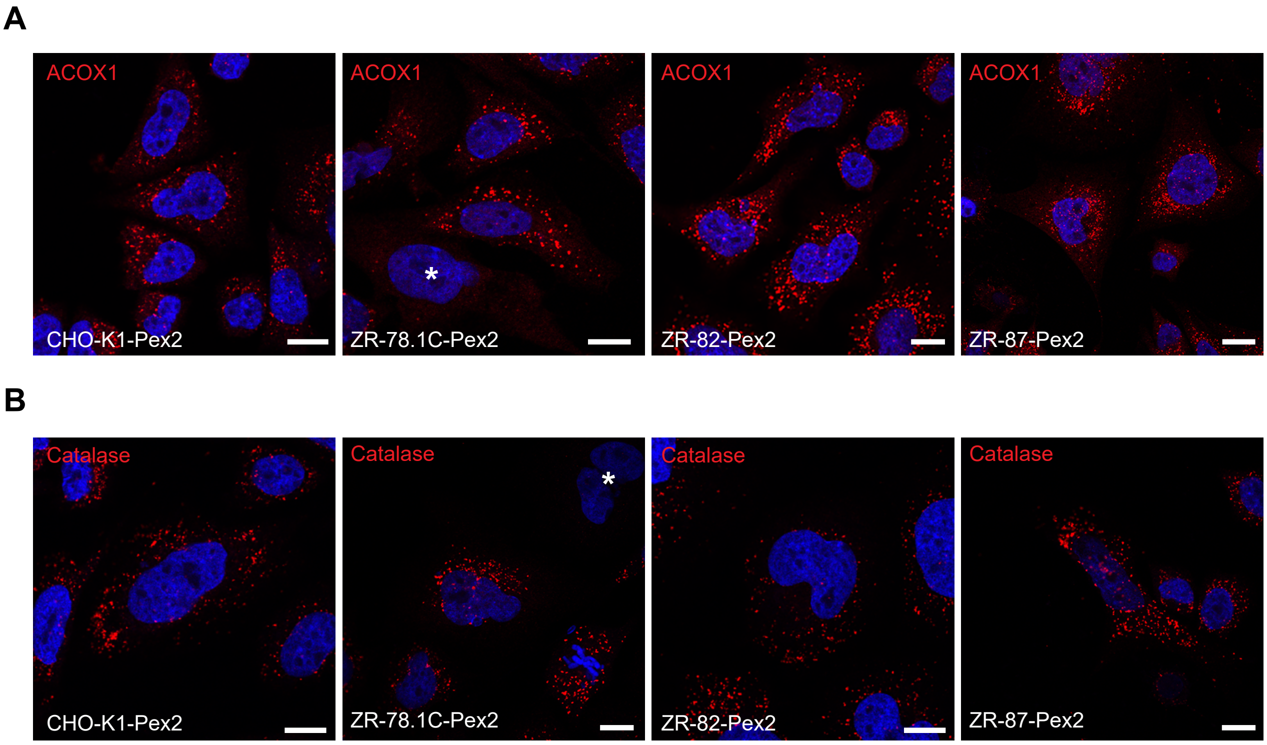

Figure S1, related to Figure 2

Figure S1, related to Figure 2. Functional peroxisomes are restored in peroxisome-deficient CHO mutants (ZR-78.1C, ZR-82, ZR-87) upon complementation with rat *Pex2* cDNA. Cells were immunostained with antibodies against the peroxisomal matrix proteins ACOX1 (**A**) and catalase (**B**). The nuclei were stained with DAPI (blue). An asterisk indicates non-transfected cells without import-competent peroxisomes. The scale bars represent 10 μm.

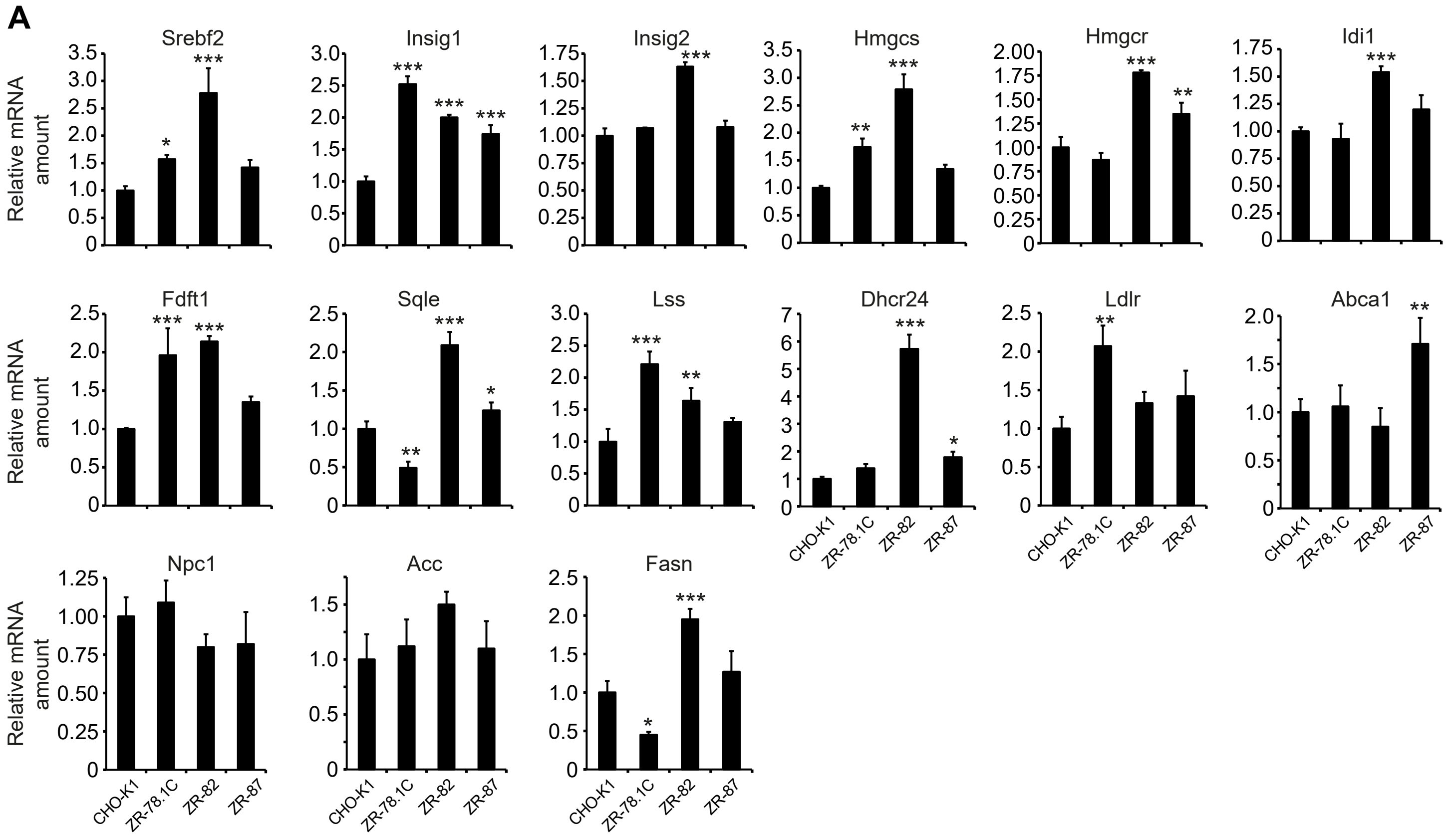

Figure S2, related to Figure 4

Figure S2, related to Figure 4. Expression of genes involved in cholesterol biosynthesis and its regulation, cholesterol efflux, and fatty acid synthesis in CHO-K1 and peroxisome-deficient CHO cells cultured in medium containing 10% FCS. Each value represents the amount of mRNA relative to that in CHO-K1, which was arbitrarily defined as 1. Data are mean ± SD (n = 3). Statistical analysis was performed using one-way ANOVA followed by Dunnett's multiple comparisons test. *, $p < 0.05$; **, $p < 0.01$; ***, $p < 0.001$ *versus* CHO-K1.

Table S1. [^{14}C]acetate incorporation into cholesterol from cells

Cell line	Cholesterol specific activity [dpm/nmol/4 h]
CHO-K1	1167 ± 181
ZR-78.1C	356 ± 33 *
ZR-82	454 ± 18 *
ZR-87	316 ± 40 *

Cells were incubated with [^{14}C]acetate for 4 h. Following saponification and petroleum ether extraction, the nonsaponifiable lipids were analyzed by reverse-phase radio-HPLC, and the specific activities of the radiolabeled products (e.g., sterols, squalene) were determined. Each value represents the average ± range (n = 2). *, $p < 0.05$ *versus* CHO-K1.

Table S2. Antibodies for immunofluorescence (IF) and western blot analysis

Target	Host	Dilution		Source
		Western	IF	(Product number, company)
PEX14	Rabbit	1:1000	1:100	10594-1-AP, Proteintech
Catalase	Rabbit	1:8000	1:500	219010, Calbiochem
ACOX1	Rabbit	0.01 µg/µl	0.06 µg/µl	Gift from A. Völkl and D. Fahimi (1)
ACBD5	Rabbit	1:1000	1:100	21080-1-AP, Proteintech
GRP78	Goat	1:500	1:75	sc-1051, Santa Cruz
GRP94	Rat	1:200		RT-102-P1, Neomarkers
FDPS	Rabbit	0.32 µg/ml		HPA028200, Atlas Antibodies
FDFT1	Mouse	1:1000		611808, BD Biosciences
HMGCR	Rabbit	1:3000		Kovacs et al., 2004 (2)
MVK	Rabbit	11 µg/ml		Kovacs et al., 2004 (2)
IDI1	Rabbit	9.5 µg/ml		Kovacs et al., 2004 (2)
γ-Tubulin	Mouse	1:5000		T6557, Sigma Aldrich
α-Tubulin	Rabbit	1:5000		ab18251, Abcam
GFP	Chicken		1:500	GFP-1020, Aves Labs
Giantin	Rabbit		1:1000	PRB-114C, Covance
pAKT (S473)	Rabbit	1:1000		#4058, Cell Signaling
AKT	Rabbit	1:1000		#9272, Cell Signaling
p-p70 S6K (T421/S424)	Rabbit	1:1000		#9204, Cell Signaling
pS6 (S235/236)	Rabbit	1:1000		#2211, Cell Signaling
S6	Rabbit	1:1000		#2217, Cell Signaling
pERK1/2 (T202/Y204)	Rabbit	1:1000		#9101, Cell Signaling
ERK1/2	Rabbit	1:1000		#9102, Cell Signaling

(1)Beier, K., Völkl, A., Hashimoto, T. and Fahimi, H.D. (1988). Selective induction of peroxisomal enzymes by the hypolipidemic drug bezafibrate. Detection of modulations by automatic image analysis in conjunction with immunoelectron microscopy and immunoblotting. *Eur J Cell Biol* 46, 383-393.
(2)Kovacs, W.J., Tape, K.N., Shackelford, J.E., Wikander, T.M., Richards, M.J., Fliesler, S.J., et al. (2004). Disturbed cholesterol homeostasis in a peroxisome-deficient *PEX2* knockout mouse model. *Mol Cell Biol* 24(1), 1-13 (2004).

Table S3. Quantitative real-time PCR primer.

Gene	Species	Forward primer	Reverse primer
Srebf2	*Cricetulus griseus*	5'-GCGGTCTGGAGACCATGGA-3'	5'-GCATAGCTGCTCTGAAAACAAATCA-3'
Insig1	*Cricetulus griseus*	5'-TCACAGTGACTGAACTTCAGGGCA-3'	5'-GAGTCTTCATCACACCCAGGAC-3'
Insig2	*Cricetulus griseus*	5'-CGAGTCACCTGGGCCCAAAAG-3'	5'-CAAGTTCAACACTAAGGCAAGGA-3'
Hmgcs	*Cricetulus griseus*	5'-CCTATGACTGCATTGGGCG-3'	5'-CCCAGACTCCTCAAACAGCTG-3'
Hmgcr	*Cricetulus griseus*	5'-CTTGTGGAATGTCTTGTAATTG-3'	5'-AGCCAAAGCAGCACATGAT-3'
Idi1	*Cricetulus griseus*	5'-ACCAGCCATCTTGATGAAAAACA-3'	5'-CAGCAACTATTGGTGAAACAACC-3'
Fdft1	*Cricetulus griseus*	5'-TCCCGATGCTGCGCAACTTTC-3'	5'-TGTCCACAAATTCCGCCATCCC-3'
Sqle	*Cricetulus griseus*	5'-TCTGATACACGGCTACATAG-3'	5'-ACTTGCCATGGTGGAAAGCAAC-3'
Lss	*Cricetulus griseus*	5'-CCCTGAACTATGTGGCTCT-3'	5'-ATAGGGTGTTGAGTCCTTCC-3'
Dhcr24	*Cricetulus griseus*	5'-AGGCAGCTGGAGAAGTTTGT-3'	5'-CCTCGCGGTTCATATAGCAATC-3'
Ldlr	*Cricetulus griseus*	5'-AAGGAGAAGGACACTGTTCC-3'	5'-ATGCTGGAGATAGAGTGGAG-3'
Abca1	*Cricetulus griseus*	5'-ATAGCAGGCTCCAACCCTGAC-3'	5'-GGTACTGAAGCATGTTTCGATGTT-3'
Npc1	*Cricetulus griseus*	5'-TGTTTGGTATGGAGAGTGTGGA-3'	5'-GTCACAGCAGAGACTAACATTG-3'
Acc	*Cricetulus griseus*	5'-TCCTGGGGAGAAAACAGAGA-3'	5'-ACGTTCTGCCTGCACTTTTT-3'
Fasn	*Cricetulus griseus*	5'-AGTTACATCATCACTGGTGG-3'	5'-TTGCTTGTTGACACTAGCAC-3'
Gapdh	*Cricetulus griseus*	5'-CGTGTCCGTTGTGGATCTGA-3'	5'-CCTGCTTCACCACCTTCTTGAT-3'
Actin	*Cricetulus griseus*	5'-AGCTGAGAGGGAAATTGTGCG-3'	5'-GCAACGGAACCGCTCATT-3'
Grp94	*Cricetulus griseus*	5'-TGGGAAGAGGTTCCAGAATG-3'	5'-CGTGAGACGCTGAGATACCA-3'
Dnajb9	*Cricetulus griseus*	5'-CACTTCCAGACACACCAGGA-3'	5'-TTCCTCTTCGCTGAGTGACA-3'
Calr	*Cricetulus griseus*	5'-ACGTGAAGCTGTTTCCGAG-3'	5'-TGTTGATCAGCACATTCTTGC-3'
Canx	*Cricetulus griseus*	5'-CCTCAGATTGCCAACCCTAA-3'	5'-AGCACTGAAAGGCGTCATCT-3'
Atf4	*Cricetulus griseus*	5'-TTCTCCAGCGACAAGGCTAAG-3'	5'-GCACTGACCAACCCATCCA-3'
Chop	*Cricetulus griseus*	5'-GGGAGCTGGAAGCCTGGTAT-3'	5'-GGGACCCCCATTTTCATCTG-3'

Gadd34	*Cricetulus griseus*	5’-CCTGGTCTGCAAAGTGCTGAT-3’	5’-CCAGCTCAGTCACTCCCTCTTC-3’
Xbp1t	*Cricetulus griseus*	5’-CTCCAGAGACGGAGTCCAAG-3’	5’-AAAGGGAGGCTGGTAAGGAA-3’
Xbp1u	*Cricetulus griseus*	5’-CTCCAGAGACGGAGTCCAAG-3’	5’-CAGAGGTGCACGTAGTCTGAGTGCTG-3’
Xbp1s	*Cricetulus griseus*	5’-CTCCAGAGACGGAGTCCAAG-3’	5’-GAGTCCGCAGCAGGTG-3’
Srebf2	*Mus musculus*	5’-GCAGCAACGGGACCATTCT-3’	5’-CCCCATGACTAAGTCCTTCAACT-3’
Insig1	*Mus musculus*	5’-CACGACCACGTCTGGAACTAT-3’	5’-TGAGAAGAGCACTAGGCTCCG-3’
Insig2b	*Mus musculus*	5’-CCGGGCAGAGCTCAGGAT-3’	5’-GAAGCAGACCAATGTTTCAATGG-3’
Hmgcr	*Mus musculus*	5’-AGCTTGCCCGAATTGTATGTG-3’	5’-TCTGTTGTGAACCATGTGACTTC-3’
Pmvk	*Mus musculus*	5’-CTTGGAGGTAACATCTGTGCTC-3’	5’-TGCTCGCATCCAGAAGTCTCT-3’
Mvd	*Mus musculus*	5’-CCGGTCAACATCGCAGTTATC-3’	5’-TTGTGGTCGTTTTTAGCTGGT-3’
Idi1	*Mus musculus*	5’-ACCAGCCATCTTGATGAAAAACA-3’	5’-CAGCAACTATTGGTGAAACAACC-3’
Fdps	*Mus musculus*	5’-GGAGGTCCTAGAGTACAATGCC-3’	5’-AAGCCTGGAGCAGTTCTACAC-3’
Fdft1	*Mus musculus*	5’-ATGGAGTTCGTCAAGTGTCTAGG-3’	5’-CGTGCCGTATGTCCCCATC-3’
Sqle	*Mus musculus*	5’-ATAAGAAATGCGGGGATGTCAC-3’	5’-ATATCCGAGAAGGCAGCGAAC-3’
Sc4mol	*Mus musculus*	5’-AAACAAAAGTGTTGGCGTGTTC-3’	5’-AAGCATTCTTAAAGGGCTCCTG-3’
Lss	*Mus musculus*	5’-TCGTGGGGGACCCTATAAAAC-3’	5’-CGTCCTCCGCTTGATAATAAGTC-3’
18S rRNA	*Mus musculus*	5’-GTTCCGACCATAAACGATGCC-3’	5’-TGGTGGTGCCCTTCCGTCAAT-3’
Ppia	*Mus musculus*	5’-GAGCTGTTTGCAGACAAAGTTC-3’	5’-CCCTGGCACATGAATCCTGG-3’
Herpud1	*Mus musculus*	5’-GCAGCCGGACAACTCTAATCA-3’	5’-CTCTTTGCCGTAAACCATCACT-3’
Edem1	*Mus musculus*	5’-AGTCAAATGTGGATATGCTACGC-3’	5’-ACAGATATGATATGGCCCTCAGT-3’
Gadd45	*Mus musculus*	5’-CCGAAAGGATGGACACGGTG-3’	5’-TTATCGGGGTCTACGTTGAGC-3’
Grp78	*Mus musculus*	5’-ACTTGGGGACCACCTATTCCT-3’	5’-ATCGCCAATCAGACGCTCC-3’
Grp94	*Mus musculus*	5’-TCGTCAGAGCTGATGATGAAGT-3’	5’-GCGTTTAACCCATCCAACTGAAT-3’
Dnajc3	*Mus musculus*	5’-GGCGCTGAGTGTGGAGTAAAT-3’	5’-GCGTGAAACTGTGATAAGGCG-3’
Pdia4	*Mus musculus*	5’-TCCCATTGCTGTAGCGAAGAT-3’	5’-GGGGTAGCCACTCACATCAAAT-3’

Ire1a	*Mus musculus*	5’-ACACCGACCACCGTATCTCA-3’	5’-CTCAGGATAATGGTAGCCATGTC-3’
Nupr1	*Mus musculus*	5’-CCCTTCCCAGCAACCTCTAAA-3’	5’-TCTTGGTCCGACCTTTCCGA-3’
Atf4	*Mus musculus*	5’-ATGGCGCTCTTCACGAAATC-3’	5’-ACTGGTCGAAGGGGTCATCAA-3’
Chop	*Mus musculus*	5’-CTGGAAGCCTGGTATGAGGAT-3’	5’-CAGGGTCAAGAGTAGTGAAGGT-3’
Gadd34	*Mus musculus*	5’-GAGGGACGCCCACAACTTC-3’	5’-TTACCAGAGACAGGGGTAGGT-3’
Atf3	*Mus musculus*	5’-GAGGATTTTGCTAACCTGACACC-3’	5’-TTGACGGTAACTGACTCCAGC-3’
Trib3	*Mus musculus*	5’-GCAAAGCGGCTGATGTCTG-3’	5’-AGAGTCGTGGAATGGGTATCTG-3’
Xbp1t	*Mus musculus*	5’-TGGCCGGGTCTGCTGAGTCCG-3’	5’-GTCCATGGGAAGATGTTCTGG-3’
Xbp1s	*Mus musculus*	5’-CTGAGTCCGAATCAGGTGCAG-3’	5’-GTCCATGGGAAGATGTTCTGG-3’
